# Supplementary material for: Perspectives of healthcare and social support sector policymakers on potential solutions to mitigate financial impact among people with TB in Mozambique: a qualitative study
Source: BMJ Open. 2023 Aug 31;13(8):e073234. doi: 10.1136/bmjopen-2023-073234 (PMC10476108; doi:10.1136/bmjopen-2023-073234)
Supplement: Supplementary data [file bmjopen-2023-073234supp001.pdf]

Coordination of HEalth and Social care for TB patients in Mozambique: Policy dialogue and situation analysis - CHEST ENGLISH_**DECISION MAKERS**

Participant ID:______________

SCREENING BY RESEARCH TEAM:

Thank you for speaking with me_________________ regarding the "**Coordination of HEalth and Social care for TB patients in Mozambique**" study. This project is being led by researchers from [Karolinska Institute from Sweden and Instituto Nacional de Saúde from Mozambique]

I would like to ask you a few questions in order to determine whether you may be eligible for the research. Before I begin the screening I would like to tell you a little bit about the research. The purpose of this research is to create evidence of feasible and locally-appropriate social support scheme and strengthen understanding of the socioeconomic impact of TB in Mozambique.

Would you like to continue with the screening? The screening will take approximately one minute. You do not have to answer any questions you do not wish to answer or are uncomfortable answering, and you may stop at any time. Your participation in the screening is voluntary. If you do not qualify for the study, your answers to the screening will be destroyed. If you qualify for the research, decide to participate and agree to the informed consent, the answers to the screening will be only accessible to the research team.

Would you like to continue?

[*If no, thank the person and conclude the interview*]
[*If yes, continue with the IFC*]

Nhassengo P, *et al. BMJ Open* 2023; 13:e073234. doi: 10.1136/bmjopen-2023-073234

Coordination of HEalth and Social care for TB patients in Mozambique: Policy dialogue and situation analysis - CHEST ENGLISH_**DECISION MAKERS**

Open- Ended Questions

Note: The in-depth interview will be open-ended and guided by the respondent's answers. This outline reflects a general guide for the in-depth interviews.

The interviews are meant to help us understand what could be the better socio economic support scheme for TB patient in Mozambique. We are particularly interested in understanding how you financial status changes after TB diagnose and what kind of social support scheme you value most. Actual questions asked during the interview will vary based on participant responses.

SCRIPT: Now I'd like to talk to you about your experience with TB social support services

BACKGROUND

1. Please tell me of your experience in this (health/social) service?
2. What are your main responsibilities?
3. What are the key decisions or activities that your service undertakes?

TUBERCULOSIS SUPPORT

4. Does your service deal with people who have tuberculosis?
   If yes, how? If no, why not?
5. Are you aware of support available to TB patients beyond medication? *Please describe. Does your organization do this? What other organizations are involved?*
6. What kind of support do you think TB patients need most?
7. What are the main challenges facing TB patients? What can/does your organization do about these?

INTERVENTIONS AND INTERSECTORAL WORK

1. To what extent does your organization work intersectorally (with another government department, health/social)?
2. Do they work together to support TB patients? If yes, how? If not, why not?
3. What would be a good way of collaborating with other departments for supporting TB patients? What could be done? Whose responsibility should it be to collaborate?
4. What kind of intervention in your opinion would be suitable to supporting TB patients economically and socially? What should they get, and whose responsibilities would this be?

Nhassengo P, *et al. BMJ Open* 2023; 13:e073234. doi: 10.1136/bmjopen-2023-073234
