# Supplementary material for: Perspectives of healthcare and social support sector policymakers on potential solutions to mitigate financial impact among people with TB in Mozambique: a qualitative study
Source: BMJ Open. 2023 Aug 31;13(8):e073234. doi: 10.1136/bmjopen-2023-073234 (PMC10476108; doi:10.1136/bmjopen-2023-073234)
Supplement: Supplementary data [file bmjopen-2023-073234supp002.pdf]

| Themes | Category | Sub-category | Codes |
|---|---|---|---|
| (1) Policymakers suggested several solutions but thought that the implementation of many of them would be limited by available resources | Benefits of supporting people with TB | – | – |
| | Incurred costs and mitigation | Cost mitigation | Coping mechanisms |
| | | | Current mitigation activities |
| | | | Provision entity |
| | | | Types of strategies for cost mitigation |
| | | Financial impact | Impact on overall economy |
| | | | Ability to cope with the TB-related cost |
| | | | Impact on the course of disease |
| | | | Impact on families' lives |
| | | | Impact on patients' lives |
| | | Type of incurred cost | During care seeking |
| | | | During treatment |
| | | | Factor influencing the occurrence of cost |
| | Potential solutions for addressing costs | Challenges faced by people with TB | Bottlenecks of TB treatments |
| | | | Problems faced by people with TB |
| | | | Problems faced by patients' families |
| | | Source of support provision | Availability of support |
| | | | Current institutional actions |
| | | Type of needed support | Better housing |
| | | | Challenges of receiving direct cash |
| | | | Children support / Education for children |
| | | | Community actors |
| | | | Easy access and expansion of good quality health service |
| | | | Home visits through deployment of health workers |
| | | | Psychological support |
| | | | Cash |
| | | | Food |
| | | | Transport |
| (2) Lack of shared views or processes related to intersectoral collaboration between health and social protection sector hinders design and implementation of social support for people with TB | Existing collaboration with other sectors | Challenges of collaborations | Barriers |
| | | | Facilitators |
| | | Existing collaborations with other sectors | Key stakeholders currently left aside |
| | | | Involved stakeholders |
| | | | Mechanisms of collaborations and referrals of people with TB |
| | | | Type of collaborating institutions |

**Table 3:** Categories and codes created during the coding process on Dedoose software, and the themes generated during subsequent analysis
