# Supplementary material for: Perspectives of healthcare and social support sector policymakers on potential solutions to mitigate financial impact among people with TB in Mozambique: a qualitative study
Source: BMJ Open. 2023 Aug 31;13(8):e073234. doi: 10.1136/bmjopen-2023-073234 (PMC10476108; doi:10.1136/bmjopen-2023-073234)
Supplement: Author's manuscript [file bmjopen-2023-073234.draft_revisions.pdf]

BMJ Open is committed to open peer review. As part of this commitment we make the peer review history of every article we publish publicly available.

When an article is published we post the peer reviewers' comments and the authors' responses online. We also post the versions of the paper that were used during peer review. These are the versions that the peer review comments apply to.

The versions of the paper that follow are the versions that were submitted during the peer review process. They are not the versions of record or the final published versions. They should not be cited or distributed as the published version of this manuscript.

BMJ Open is an open access journal and the full, final, typeset and author-corrected version of record of the manuscript is available on our site with no access controls, subscription charges or pay-per-view fees (http://bmjopen.bmj.com).

If you have any questions on BMJ Open's open peer review process please email info.bmjopen@bmj.com

BMJ Open

Perspectives of healthcare and social support sector policymakers on potential solutions to mitigate financial impact among people with TB in Mozambique: a qualitative study

Journal:	*BMJ Open*
Manuscript ID	bmjopen-2023-073234
Article Type:	Original research
Date Submitted by the Author:	27-Feb-2023
Complete List of Authors:	Nhassengo, Pedroso; Instituto Nacional de Saúde; Karolinska Institute, Department of Global Public Health<br>Yoshino, Clara; Karolinska Institute, Department of Global Public Health; Health and Social Protection Action Research and Knowledge Sharing Network<br>Zandamela, Américo; Instituto Nacional de Saúde<br>De Carmo, Verónica; Instituto Nacional de Saúde<br>Burström, Bo; Karolinska Institutet, Department of Global Public Health<br>Khosa, Celso; Instituto Nacional de Saúde<br>Wingfield, Tom; Liverpool School of Tropical Medicine, Department of International Public Health and Clinical Sciences; Karolinska Institute, Department of Global Public Health<br>Lönnroth, Knut; Karolinska Institute, Department of Global Public Health; Health and Social Protection Action Research and Knowledge Sharing Network<br>Atkins, Salla; Tampere University, New Social Research and Faculty of Social Sciences; Karolinska Institutet, Department of Global Public Health
Keywords:	Tuberculosis < INFECTIOUS DISEASES, Health policy < HEALTH SERVICES ADMINISTRATION & MANAGEMENT, PUBLIC HEALTH

SCHOLARONE™
Manuscripts

I, the Submitting Author has the right to grant and does grant on behalf of all authors of the Work (as defined in the below author licence), an exclusive licence and/or a non-exclusive licence for contributions from authors who are: i) UK Crown employees; ii) where BMJ has agreed a CC-BY licence shall apply, and/or iii) in accordance with the terms applicable for US Federal Government officers or employees acting as part of their official duties; on a worldwide, perpetual, irrevocable, royalty-free basis to BMJ Publishing Group Ltd ("BMJ") its licensees and where the relevant Journal is co-owned by BMJ to the co-owners of the Journal, to publish the Work in this journal and any other BMJ products and to exploit all rights, as set out in our licence.

The Submitting Author accepts and understands that any supply made under these terms is made by BMJ to the Submitting Author unless you are acting as an employee on behalf of your employer or a postgraduate student of an affiliated institution which is paying any applicable article publishing charge ("APC") for Open Access articles. Where the Submitting Author wishes to make the Work available on an Open Access basis (and intends to pay the relevant APC), the terms of reuse of such Open Access shall be governed by a Creative Commons licence – details of these licences and which Creative Commons licence will apply to this Work are set out in our licence referred to above.

Other than as permitted in any relevant BMJ Author's Self Archiving Policies, I confirm this Work has not been accepted for publication elsewhere, is not being considered for publication elsewhere and does not duplicate material already published. I confirm all authors consent to publication of this Work and authorise the granting of this licence.

For peer review only

Title: Perspectives of healthcare and social support sector policymakers on potential solutions to mitigate financial impact among people with TB in Mozambique: a qualitative study

Authors: *Pedroso Nhassengo[1,2,3], Clara Yoshino[2,3], Américo Zandamela[1], Verónica De Carmo[1], Bo Burström[2,3] Celso Khosa[1], Tom Wingfield[2,3,4,5], Knut Lönnroth[2,3], Salla Atkins [2,3,6]*

Affiliations:

[1] Instituto Nacional de Saúde, Marracuene, Mozambique
[2] Department of Global Public Health, Karolinska Institutet, Sweden
[3] Health and Social Protection Action Research and Knowledge Sharing Network (www.sparksnetwork.ki.se), Stockholm, Sweden
[4] Departments of International Public Health and Clinical Sciences, Liverpool School of Tropical Medicine, the UK
[5] Tropical Infectious Diseases Unit, Liverpool University Hospital NHS, Foundation Trust, Liverpool, UK
[6] Health Sciences, Faculty of Social Sciences, Tampere University, Finland

Corresponding Author:
Pedroso Nhassengo
PO box: 264
Email address: pedroso.nhassengo@ins.gov.mz
Word Count: 4920

For peer review only

ABSTRACT

Introduction: Tuberculosis (TB) is a communicable disease that continues to be a major cause of illness and one of the leading causes of death worldwide. People in lower socioeconomic strata are at increased risk of developing TB and being pushed deeper into poverty. Minimising TB-related financial impact is vital to reduce poverty but requires broader actions than are achievable by the healthcare sector alone. We aimed to understand healthcare and social support sector policymakers' perspectives on potential solutions to mitigate financial impact among people with TB and their households in Mozambique.

Methods: This was a qualitative study with primary data collection through semi-structured one-to-one in-depth interviews with policymakers in health and social support sector in Gaza and Inhambane provinces. A total of 27 participants were purposefully sampled. Participants were asked about their perspectives on TB-related financial impact and potential solutions to mitigate such impact. Data were analysed using content analysis.

Results: Participants reported that people with TB are not explicitly included in existing social support policies because TB *per se* is not part of the eligibility criteria. People with TB and underweight or HIV were enrolled in social support schemes providing food or cash. Two themes were generated from the analysis: (1) Policymakers suggested several mitigation solutions but thought their implementation would be limited by available resources; and (2) Lack of shared views or processes related to intersectoral collaboration between health and social support sector hinders design and implementation of social support for people with TB.

Conclusion: Despite health and social sector policymakers reporting a willingness for intersectoral collaboration to mitigate TB-related financial impact, current approaches were perceived to be unilateral. Collaboration across sectors should focus on working together to improve the existing social support programmes to mitigate TB-related financial impact.

Keywords: Tuberculosis, financial impact, social support, poverty, intersectoral collaboration, Mozambique.

For peer review only

INTRODUCTION

Tuberculosis (TB) is a leading cause of death due to infection, killing 1.6 million people globally[(1,2)]. Mozambique, which reported 98,000 TB cases in 2021, is on the WHO list of high-TB burden countries that collectively contribute 86% of the global TB burden[(2,3)]. The Mozambican health system faces several challenges in addressing this burden including low health provision coverage, low rates of case detection and lack of laboratories for drug susceptibility testing[(2,4)]. The World Health Organisation's (WHO) 2015 "End TB Strategy" aimed for zero TB-affected households to be facing catastrophic costs (defined as total cost exceeding 20% of annual household income) by the year 2020[(5)] and only 52% of TB-affected households were protected by 2021[(2)]. This focus on the importance of social support and protecting vulnerable populations was reinforced in the Sustainable Development Goal 3.3[(6)] and the United Nations High-Level Meeting on TB (UNHLM-TB)[(7, 8)].

In many settings, people with TB incur costs equivalent to more than half of their household's annual income due to TB illness and care seeking[(9,10)]. Financial impact due to TB and drug resistance (DR-TB) has been found to be independently associated with adverse TB treatment outcomes (death during treatment, loss to follow up, treatment failure or recurrent TB disease within 30 months of starting TB treatment)[(11,12)]. People from low socioeconomic strata or those with DR-TB are at a particularly high risk of severe financial impact[(11,10)]. Therefore, there is a growing consensus that progress towards the "End TB strategy" targets that will require not only investment in strengthening biomedical responses through improving access and effectiveness of health care delivery[(13)] but also actions on the socio-economic determinants and consequences of TB, as well as measures to improve quality of life and wellbeing after TB-episode[(11,14,5)], especially in Low- and Middle-Income Countries (LMIC). The holistic impact of TB on wellbeing, finances, and quality of life requires simultaneous provision of biomedical and socioeconomic approaches to control TB, which in turn requires intersectoral collaboration[(15,16)] especially between healthcare and social sectors[(17)]. However, despite a high proportion of people with TB and their households being economically vulnerable and likely to benefit significantly from social support, they are rarely covered by social support policies.

In Mozambique as in many other settings, TB disease, despite its severe consequences for people and families affected by it[(18)], is not a formal criteria of eligibility for social support. To begin developing potential for such support requires collaboration between different sectors. To date, however, there is little knowledge about how a collaboration or pathway between the healthcare and social support sector would, or could, work in practice in Mozambique. Our study therefore focuses on understanding the current landscape of collaboration, and potential for future collaboration.

The theory underlying this study is that although intersectoral collaboration is complex and sometimes hard to achieve, it will be one of the fundamental issues to consider and address in order to mitigate financial impact of TB-affected households[(19)]. Common goals, mutual trust and commitment to share the risks, allocation of time and resources are unquestionable preconditions for a sustainable collaboration[(19,20,21)]. Other dimensions to be accounted for include the scope and type of collaboration, efficiency of the collaboration processes and management of the activities and interaction between collaborating parties, which encompasses

For peer review only - http://bmjopen.bmj.com/site/about/guidelines.xhtml

communication and motivation to collaborate[19]. The role of intersectoral collaborations to address the financial impact faced by people with TB and their households have not previously been studied in Mozambique. We aimed to understand healthcare and social support sector policymakers' perspectives on potential solutions to mitigate financial impact among people with TB and their households in Mozambique, a low-income high TB burden country.

METHODS

Study design

This was a qualitative study with primary data collection using semi-structured one-to-one in-depth interviews with policymakers either in healthcare or in social support sectors. Patients or the public were not involved in the design, conduct, reporting, or dissemination plans of our research.

Setting

Mozambique is a Sub-Saharan African country with an estimated population of 31 million people[23], a literacy rate of 61%, and 63.7% living on less than $1.90 a day[24]. Study participants were recruited in seven districts of Inhambane and Gaza provinces in Mozambique (Figure 1). These provinces had 28 districts that jointly contributed to 12.8% of the country's TB notifications in 2021[25]. Since all 28 districts met the study site inclusion criteria, we randomly selected two districts in each province considering the type of districts and geographical representation and, purposely included the provincial representations of both sectors as they present distinct socioeconomic landscapes and Vilanculos as it shared social sector representation with Inhassoro (a selected district). All other selected districts had both health and social sector representation.

Structure of healthcare and social protection system

The Mozambican public healthcare services encompasses four levels (figure 2). The lower level provides primary healthcare including free-of-charge TB services (diagnosis and treatment)[4] and initial assessment for social support through the Social Action Sector. The Social Support services are delivered by the National Institute of Social Action (INAS) with the aims of preventing situations of deprivation and promoting the social integration of vulnerable groups through social support following four operational programmes namely: **i) Basic Social Subsidy Programme (PSSB)** providing regular unconditional cash transfer to vulnerable people (mainly people older than sixty and people with disabilities); **ii) Direct Social Support Programme (PAS-D)** providing food baskets to vulnerable people and households including people with chronic illnesses; **iii) Productive Social Action Programme (PASP)** providing temporary employment in urban and rural areas and **iv) Social Services Action Programme (PROSAS)** providing direct support to users of social care units that provides daily meals to elderly people, homeless and orphan in specific places [25,26]. There

For peer review only - http://bmjopen.bmj.com/site/about/guidelines.xhtml

is no social support scheme available specifically targeted towards TB-affected people and their households. Both healthcare and social support sectors have paper-based registration.

Study participants and data collection

The participants were purposefully recruited in the selected districts between October 2021 and February 2022 using snowballing and maximum variation sampling[28]. The research team compiled a list of work positions of interest in both sectors. Pre-recruitment was conducted either by email or phone. A total of 34 people were invited to participate, however, 7 were not able to participate due to personal reasons and two of those were replaced by an equivalent respondent. Eligibility criteria were (1) age 18 years or older, (2) with at least one year of active work in position and (3) capable of providing informed consent. Informed consent and data were collected face-to-face by the first author (PN). Interviews were conducted in a quiet and private place at participant's offices and lasted between 30 to 90 minutes. We stopped data collection with 27 participants when we considered data to be saturated[29]. Interviews were conducted following a semi-structured topic guide that covered three major sections: i) perceptions of financial impact faced by people with TB; ii) potential solutions to mitigate the financial impact; and iii) barriers and facilitators of intersectoral collaborations. Interviews were audio-recorded, transcribed *verbatim* and translated into English. Transcriptions were controlled for quality against the recordings by Research Assistants (Ras) and against translations by PN. Non-verbal communication and other important information were captured as field notes during each interview.

Of a total of 27 participants enrolled, 14 (52%) were from Gaza and 13 (48%) were from Inhambane provinces. The overall median age was 37 (Interquartile Range, IQR: 32 - 47) years old. Most respondents were male (56%) and median time at the working position was four years (IQR: 3 – 5). About three quarters of participants had completed a university degree (Table 1).

**Demographic Characteristics**	**Gaza n=13**	**Inhambane n=14**	**Total n=27**
**Median Age (IQR)**	**33 (32-45)**	**39 (33-48)**	**37 (32-47)**
**Gender n(%)**			
Male	7 (53.9)	8 (57.1)	15 (55.6)
Female	6 (46.2)	6 (42.7)	12 (44.4)
**Work Position n(%)**			
Healthcare policymakers	**8 (61.5)**	**8 (57.1)**	**16 (59.3)**
District/clinical directors	3 (23.1)	2 (14.3)	5 (18.5)
Public health/TB focal points	5 (38.5)	6 (42.9)	11 (40.7)
Social Support policymakers	**5 (38.5)**	**6 (42.9)**	**11 (40.8)**
Programme managers	0 (0)	2 (14.3)	2 (7.4)
Social support focal points	4 (30.8)	3 (21.4)	7 (25.9)
District directors	1 (7.7)	1 (7.1)	2 (7.4)

For peer review only - http://bmjopen.bmj.com/site/about/guidelines.xhtml

**Median Time in Work Position in years (IQR)**	**4 (3-5)**	**3.5 (2-4)**	**4 (2-5)**
**Education n(%)**			
Less than Secondary School	0 (0)	0 (0)	0 (0)
Secondary School	5 (38.5)	3 (21.4)	8 (29.6)
University	8 (61.5)	11 (78.6)	19 (70.4)

Table 1. Participants' demographic characteristics

Data Analysis

Qualitative content analysis was adopted as proposed by Graneheim and Lundman[30]. Data were deductive and inductively coded by PN and CY using Dedoose software and analysed by PN, CY and SA. Three interviews were double coded to verify the similarity of the encodings. For independent coding, the researchers had regular discussions about the understanding of the codes. The deductive coding was based on the topic guide and preliminary analysis of the interviews. This was followed by an inductive coding with the inclusion of codes that emerged from the data during the deductive coding. The interview text was sorted into four content areas: i) incurred cost and mitigation; ii) types of support for people with TB; iii) benefits of supporting people with TB and iv) intersectoral collaboration. Texts corresponding to these areas were extracted from the interview transcriptions and brought together into one text, which formed the unit of analysis. The text was divided into meaning units that were condensed. The condensed meaning units were, abstracted and labelled with a code. Through a manifest analysis, relevant codes to the research questions were rearranged and grouped into categories (Table 2). The last stage consisted of interpreting the data through a latent analysis and developing the themes. Initially, three themes were developed, but after discussion one theme was excluded because it did not correspond to or answer the research questions. Full coding process is presented in the Supplementary File.

**Theme**	**Policymakers suggested a number of solutions, but thought that the implementation of many of them would be limited by available resources**			**Lack of shared views or processes related to intersectoral collaboration between health and social protection sector hinders design and implementation of social support for people with TB**		
**Category**	Potential solutions for addressing costs			Existing collaboration with other sectors		
**Codes**	Food, Cash and transport	Mobile brigades and expansion of quality healthcare system	Better housing and psychologic al support	Mechanisms of collaboration	Involved stakeholders	Type of collaborating institutions

Table 2. Example of coding

Researcher characteristics and reflexivity

The Interviews were conducted by PN. He is a medical doctor and PhD candidate with seven-years of experience in qualitative research. PN's research is based in Mozambique, and he is familiar with the health and social services system. Coding was conducted by PN and CY, who are both native Portuguese speakers and fluent English speakers. CY is a social scientist and public policy researcher. CY and SA have experience in qualitative and international social protection systems.

PN was aware that coming from a central level public institution with credentials to conduct the research at provincial level might be seen as auditing or monitoring and thus could lead participants to share only what would sound as acceptable opinions. PN spent considerable time explaining the study, obtaining voluntary informed consent and clarifying participants' doubts. The interview began only when the rapport and trust was built between PN and the participants.

CY's experiences of similar health and social services from Brazil added value to the analysis. She immersed herself in the data and got a thorough understanding of its the content and context. The team's experience on TB and social protection in Africa and globally improved the understanding of the research findings.

Ethical considerations

All participants were provided detailed information about the study and provided a written informed consent before the interview**.** The study was performed in accordance with the study protocol, the Declaration of Helsinki (last update: October 2013), was approved by the Institutional Ethics Committee of the *Instituto Nacional de Saúde* in Maputo, Mozambique (Ref. no. 001/CIBS-INS/2020) and by the Swedish Ethical Review Authority in Stockholm, Sweden (Ref. no. 2022-03297-01). Interviews were anonymized to protect participant's identity.

RESULTS

Perceptions of financial impact faced by people with TB and its consequences

Healthcare and social sector policymakers had different understanding of the need and provision of support for people with TB. Social sector's policymakers had little knowledge of the treatment and care pathways of people with TB and thus their need for support. On the other hand, healthcare policymakers were aware of the impact of TB on people's lives, and the socioeconomic implications of treatment. They reported that TB-related costs were high for people with TB and their households, and this was worse when the Primary Income Earner (defined as the person with the largest economic contribution to secure the family's basic needs) was affected or the person had DR-TB. Incurred costs due to transportation and food were reported to be a particularly troubling issue during the initial two month (intensive treatment phase). More remote areas without health facilities providing TB care, irregular availability of

For peer review only - http://bmjopen.bmj.com/site/about/guidelines.xhtml

transportation and income loss seemed to be the major causes of negative financial impact of TB.

Theme 1: Policymakers suggested a number of solutions, but thought that the implementation of many of them would be limited by available resources

Current availability and source of social support provision

The current availability of support for people with TB was seen as inadequate by both healthcare and social sector policymakers. Some healthcare policymakers reported that people with TB used to receive food supplements from the World Food Programme (WFP) but this was discontinued. They also mentioned that current support was provided by different organisations, such as the Carmelo health facility run by the catholic church in Chókwè district. While institutional actions in the healthcare sector included active tracing by community workers and decentralization of TB services to remote areas, social protection sector provided food and monetary support through PAS-D and PSSB. Beyond TB services related measures, healthcare sector could not recount other support. Both sectors mentioned the potential benefit of psychological support to people with TB and their families focusing on the disease, treatment of TB and, enrichment of food through practical mentoring about their diet. This was perceived as having positive effects including decreasing the need for support and demand for supplements at health facilities.

Community centred strategies and expansion of TB Services

Healthcare policymakers described extensively the community-based approaches that they perceived to reduce direct and indirect out-of-pocket medical costs. They mentioned, for example, brigades, which are a group of healthcare workers whose responsibilities can include community contact tracing, community based Directly Observed Therapy (DOTc), TB screening and sample collection, home visits and follow-up of people under treatment. Community-based strategies were seen as key to people with TB mainly due to reduction of travelling costs.

> *"One of our ways to reduce the cost [of people with TB] is the DOTc strategy, which is community DOT where we have our activists there. […] this way we reduce the cost of [people with TB] who have to come to the health facility twice a month." (Male, healthcare policymaker, 3 years in position)*

Expansion of healthcare services to more remote areas was considered by healthcare policymakers as a suitable strategy to reduce transportation cost and waiting time at the health facilities. They reported the expansion of TB treatment sites having as example the antiretroviral therapy (ART) services, which was expanded to all health facilities and resulted in perceived reduction of transportation cost. They also suggested that activists (staff dedicated to counselling at health facilities or at communities) and health technicians (staff with basic medical training and abilities to diagnose and prescribe medications for the most common

For peer review only - http://bmjopen.bmj.com/site/about/guidelines.xhtml

diseases) should work together as community health workers. The current approach for community work was seen as inappropriate as the healthcare sector was mainly represented by activists who lack clinical knowledge. On the other hand, health technicians or community health workers were perceived to be cheaper than medical doctors.

Food provision and monetary support for people with TB

According to both healthcare and social sector policymakers, the existing policy on provision of social support for people with TB was conditional on having other comorbidities such as HIV and underweight and, they were eligible to receive food supplements, as part of a collaboration with the nutrition programme.

From the social sector's side, people on ART or chronically ill (illnesses requiring long-term care and support) received a basic food basket containing nutritional supplementation through the Direct Social Support Programme (PAS-D). The programme initially covers six months of continuous support after which recipients are re-assessed regarding the need for continued support. If so, the support could be extended for additional six months or the recipient would be redirected to the Basic Social Subsidy Programme (PSSB), which offers lifelong monetary support. However, having TB was not *per se* an eligibility criterion for enrolling in this programme.

One social sector policymaker (Female, social sector policymaker, 3 years in position) reported that people with chronic illnesses used to receive food baskets, but due to lack of funds, they started to receive cash, as they have a lower cost compared to PAS-D food baskets. They also mentioned that there is a direct cash support for transport. However, people with TB face difficulties in receiving such support, as it requires them to provide proof of transport payment, which is seldom available on public transportation.

Barriers and facilitators of addressing TB-related costs

Participants reported different strategies undertaken by both institutions and households to mitigate TB-related financial impact. Healthcare policymakers reported strategies such as obtaining additional income by selling livestock and spouses whose husband with TB was primary income earner taking over financial responsibilities.

Cost mitigation activities conducted by both policymakers included institutionalised and non-institutionalised actions. Institutionalised actions were defined as activities implemented according to the operational procedures and non- institutionalised actions were defined as activities or actions implemented by policymakers' instant initiative. The latter resulted from participants being sensitised by the situation of the person with TB and helping them using their own resources. These consisted of different levels of involvement, from healthcare policymakers advising people with TB on possible ways to obtain some income to mobilising the community to donate food or money. Social sectors' policymakers also reported trying to enrol people with TB in any social protection programme.

For peer review only - http://bmjopen.bmj.com/site/about/guidelines.xhtml

Strategies to mitigate TB-related financial impact were reported by healthcare policymakers as still insufficient, as people with TB continue to incur high costs and therefore risk to abandon the treatment.

Potential solutions for addressing TB-related costs

Both healthcare and social sector policymakers thought that food was the most important social support option for people with TB, because the need for food cannot be compensated through other means, and many people with TB were food insecure. Lack of transport, for example, could be replaced by walking in the absence of cash; but food is a staple that is always needed by people with TB. Healthcare policymakers also reported that food was the most common form of support that people with TB asked for. However, food management was considered complicated, as it requires more complex logistics (purchase, storage, and distribution).

> *"I would prioritise food because we end up having other alternatives for transport but for food, we don't have any measure that helps us to minimise the situation of [people with TB], so they end up coming on foot if they ate that day" (Male, healthcare policymaker, 6 years in position).*

According to policymakers, the lack of food could be countered either by direct provision of food or by provision of cash. However, healthcare policymakers were reluctant to give cash due to the perceived risk of misuse, such as alcohol consumption. Despite this, social sector reported that cash provision would be practically and logistically easier and that most people would adequately use the cash, as they were instructed during psychological support. Both healthcare and social sector policymakers seemed to believe that addressing only other financial burdens, such as transportation cost, is not enough if food was not provided.

> *"[...] money, as such, I don't think so. Money creates problems ahead, that's why I am not suggesting money support. A basic food basket is better because when we give money to certain people who are undergoing treatment when they start to improve instead of buying food, they deviate the money." (Male, healthcare policymaker, 3 years in position).*

Theme 2: Lack of shared views or processes related to intersectoral collaboration between health and social protection sector hinders design and implementation of social support for people with TB.

Barriers and facilitators to collaboration between healthcare and social sectors

Participants perceived involvement of key stakeholders as an important step towards appropriate cost-mitigation strategies. The Ministry of Health, through its provincial and district representations and the Ministry of Gender, Children and Social Action, through INAS

and its provincial and district representations are considered the most important stakeholders. Other governmental stakeholders played complementary roles. The community and churches providing informal social support, were also considered important players. Participants also suggested that all stakeholders working in similar areas or in philanthropic projects should be engaged to strengthen the collaborations.

Support for people with disabilities or chronic illness was predominant in the collaboration between the healthcare and social institutions. Both healthcare and social sector participants mentioned that there was an ongoing intersectoral collaboration for supporting people with chronic illnesses. A substantial barrier was seen as the non-inclusion of TB disease in Mozambican social support policies which prevented direct provision of social support to people with TB. An alternative measure was registering people with TB according to other chronic illness or disability that they might have such as chronic lung disease or malnutrition. Overall, healthcare policymakers seemed dissatisfied with the current collaboration. Waiting time for support to be provided, poor communication, perceived lack of resources and cumbersome vulnerability assessment were reported as being the source of dissatisfaction. They described waiting time for support provision as long and inadequate for people with TB and opined that often the social sector counterpart delayed the feedback of support applications.

> *"The most obvious barrier is the delay in response [...] because the feedback of applications take time. At some point, we get the response while the patient has already finished the treatment or maybe others have already abandoned or died." (Female, healthcare policymaker, 33 years in position).*

Participants from the healthcare sector seemed to not understand how the social sector proceeds for support allocation. Information about the correct steps as well as the eligibility for support provision seemed to be scarce. On the other hand, social sector assists very few people with chronic illness (including TB), compared to elderly people and children. This may be explained by limited dissemination of the programmes, lack of understanding and low referral rates by the health sector. Additionally, both participants reported resources limitation to cover new applications. Allocated budget and goals were pre-defined at central level and new beneficiaries entered the programmes only when current beneficiaries were discharged from it resulting in long waiting list.

On top of this, the lifelong support provided to beneficiaries with chronic illnesses was perceived to be a small amount varying according to the number of household members (from $8 to 16 per month). Almost all participants agreed that the amount was insufficient to handle basic needs even for a single-person household.

Participants from social sector stressed that the sector, understandably, lacks clinical knowledge or competence. Participants reported that a health technician or a psychologist allocated to their offices would improve the identification of sick beneficiaries at the community level and promptly refer them to the healthcare sector for further management as appropriate.

For peer review only - http://bmjopen.bmj.com/site/about/guidelines.xhtml

Intersectoral communication was raised as an area for improvement by healthcare sector but not social sector participants. One healthcare participant suggested that the intersectoral communication could be more informal and formalities could be left to a later stage and that high-level intersectoral policymaking meetings and discussions could be of benefit to define common goals and consider how best intersectoral collaboration could successfully be implemented and achieved.

Mechanisms of collaboration between healthcare and social sectors

The roles of the two major players in supporting people with TB seemed clear to all participants. The health sector's responsibility was to diagnose the TB disease, check clinical eligibility and pre-assessment of vulnerability, which enabled them to refer these people to the Social Action Sector. On the other hand, the Social Action Sector should do a more complete and detailed assessment of the beneficiary's vulnerability and then submit the application to social sector at INAS. The expectations were that INAS would provide approval for enrolment and support provision if the budget was available. Another direction of collaboration was related to how INAS' beneficiaries reach the health sector when they become sick (people with presumptive TB for example). This was unclear and may not be in place as INAS' lack of competence to assess health status limited them to counselling and advising people to go to the health facilities for better assessment and diagnosis.

DISCUSSION

This study revealed healthcare and social sector policymakers' perceptions of the financial impact and challenges faced by people with TB in Mozambique as well as current and potential joint mitigation strategies between the two sectors. Since TB is not considered an eligibility criterion in current Mozambican social support policies, people with TB and their households are not covered by adequate and equitable social support. Our findings provide valuable information about both the current provision of social support and gaps in support for people with TB, suggested solutions for support provision during treatment phase and dynamics of intersectoral collaboration between healthcare and social support sector.

People from low socioeconomic strata are disproportionately affected by TB, and our interviewees were clear, at least in the healthcare sector, that social determinants are the drivers of TB in Mozambique. Similar to other studies, there were vulnerabilities among people with TB that included poverty, low income, food insecurity, undernutrition[(31)] and risk of income loss. This was due to their livelihoods being based predominantly on farming, self-employment or informal work, with many unable to benefit from stable labour activities[(32)], health and sickness insurance coverage. This financial situation meant an inability to afford the costs associated with TB. Studies in Africa and Asia also reported similar income and job-related problems[(33)]. Distance to health facilities and its related transportation costs were perceived as negatively impacting health seeking behaviour, leading to delays in presenting at health facilities, worsening of health condition and risk of spreading the disease to cohabiting

For peer review only - http://bmjopen.bmj.com/site/about/guidelines.xhtml

people[9]. Health system-related delays in diagnosis also contribute to increasing the financial impact due to costs with transportation and out-of-pocket payments of medication taken during waiting time. We also found that healthcare policymakers thought that the TB-related costs and consequences were exacerbated by rigid DOT routines. Most people with TB struggle to have enough food and money for daily transportation to DOT sites[33,34,35]. Distance and transportation cost to the DOT site, costs of food, out-of-pocket medication and income loss were the leading causes of high costs during treatment and lack of transportation money during TB treatment was associated with missing DOT visits and TB doses and lack of food with treatment failures[10].

Although lack of food and money impacted negatively the TB treatment, food provision was the most important due to its impact on treatment adherence and medication intake. Similar studies showed that food support was the most important for more easily averting the challenges faced by people with TB[34]. However, our findings indicate that food provision requires a more complex logistical apparatus for purchasing, storage, and distribution to beneficiaries making the cash preferred over food support. The impact of existing cash support schemes was reported to be limited by low cash amounts provided. Psychological support currently provided by healthcare and social support sectors to people with TB and their household members was considered indispensable, but insufficient by itself. A study by Ahuja et al. in western India[16] showed that family support to people with TB improved their ability to remain in care. However, South African evidence suggests that while family can be an important support structure, as their resources are limited, people with TB are left without important sources of support.

We found that there were existing collaborations between two key players that could improve referral of and support for people with TB and their households: healthcare and social support sector. However, lack of alignment of aims and goals and suboptimal communication goals between the healthcare and social support sector was likely to have been influenced by lack of official collaboration establishment, definition of roles of the parties, specific action plan to be carried out, benefits and necessary resources[19]. Participants from social support sector seemed to lack knowledge about TB and its consequences beyond health, which partially explains why many of these participants did not understand why people with TB may be socioeconomically vulnerable. However, most participants mentioned that their work was done according to guidelines designed at higher levels. Thus, despite participants from both sectors reported being willing to work together to improve the collaboration, actions at the policy-making level are required.

STRENGTHS AND LIMITATIONS

Strengths of this study included good representation of policymakers both in terms of provinces and sectors, high quality and depth of interviews, and data collection conducted by the first author in his native language and with contextual experience and understanding. This allowed a comprehensive understanding of the responses and quality control of the transcripts in Portuguese and its translation to English. Additionally, data collection tools were adapted

For peer review only - http://bmjopen.bmj.com/site/about/guidelines.xhtml

during the process following the principles of adaptability, flexibility, and pragmatism [37]. An important limitation of this study was the timing of the data collection, which coincided with the period when state servants usually go for annual leave. Because of this, some potential study participants whose positions where pre-listed by the RA, could not be reached. In such situation, interview was made with the person responding for the position at data collection period. Despite this, our findings are consistent with existing international literature discussing solutions of TB-related costs[39,40]

CONCLUSIONS

TB disease is still perceived as a disease of poverty, driven by social determinants and with severe socioeconomic consequences. Our findings from key health and social sector policy makers suggest that the two sectors are willing to collaborate beyond the current modest and unilateral efforts to mitigate the financial hardship of TB. Collaboration across sectors should be strengthened and eligibility criteria for social support should be revised to explicitly include people with TB to enable mitigation of costs of care and compensation of foregone income. Lack of financial resources hampers implementation of several identified strategies that could potentially avert the TB-related cost faced by people with TB and their households. Further research is needed to explore reasons of non-inclusion of TB patients as eligible beneficiaries, as well as barriers for broader policy changes for social protection inclusion of people with TB.

ACKNOWLEDGMENTS

The authors express their gratitude to the Ministry of Health and the Ministry of Gender, Children and Social Action and study participants for their participation and support. We also would like to thank the National Tuberculosis Programme for its contribution in setting up the study. Lastly, we would like to thank Dr Kerry Viney for her support in designing the study and reviewing the paper.

FUNDING STATEMENT

This study was funded by Vetenskapsrådet through the Swedish Research Council, VR 2017-05497. TW is supported by grants from the Wellcome Trust, UK (209075/Z/17/Z), the Medical Research Council, Department for International Development, and Wellcome Trust (Joint Global Health Trials, MR/V004832/1), and a Dorothy Temple Cross Tuberculosis International Collaboration Grant from the Medical Research Foundation (MRF-131-0006-RG-KHOS-C0942), UK.

For peer review only

AUTHORS' STATEMENT

Fund acquisition and study design: Salla Atkins, Pedroso Nhassengo, Celso Khosa, Tom Wingfield, Bo Burström and Knut Lonnröth
Data collection: Pedroso Nhassengo, Clara Yoshino, Américo Zandamela, Verónica De Carmo, Bo Burström, Celso Khosa, Tom Wingfield, Knut Lönnroth, Salla Atkins
Data analysis: Pedroso Nhassengo, Clara Yoshino, Américo Zandamela, Verónica De Carmo and Salla Atkins
Writing of the first draft: Pedroso Nhassengo, Clara Yoshino, Américo Zandamela, Verónica De Carmo and Salla Atkins
Review and editing of the manuscript: Pedroso Nhassengo, Clara Yoshino, Américo Zandamela, Verónica De Carmo, Bo Burström, Celso Khosa, Tom Wingfield, Knut Lönnroth, Salla Atkins
All authors read and approved the final version of the manuscript.

For peer review only

REFERENCES

1. Organization WH. Global Tuberculosis Report 2020. Geneva; 2020.
2. World Health Organization. Global Tuberculosis report. Geneva; 2022.
3. World Health Organization. WHO global lists of high burden countries for tuberculosis (TB), TB / HIV and TB (MDR / RR-TB). 2021;2021–5.
4. Saúde M da. Avaliação e manejo de patientes com TB, Normas Nacionais, Maputo. 2019;
5. World Health Organization. Implementing the End TB Strategy: The Essentials. 2015;1–130.
6. United Nations. UN General Assembly, Transforming our world : the 2030 Agenda for Sustainable Development, 21 October 2015, A/RES/70/1, available at: https://www.refworld.org/docid/57b6e3e44.html [accessed 28 November 2019]. 2015;16301(October):1–35.
7. United Nations. Draft resolution submitted by the President of the General Assembly: scope, modalities, format and organization of the high-level meeting on the fight against tuberculosis [Internet]. New York, NY: United Nations; 2018. Available from: https://undocs.org/en/A/72/L.40
8. Stop TB Partnership. UNHLM on TB: key targets and commitments. Geneva, Switzerland: STOP TB Partnership; 2020.
9. Barter DM, Agboola SO, Murray MB, Bärnighausen T. Tuberculosis and poverty: the contribution of patient costs in sub-Saharan Africa – a systematic review. BMC Public Health. 2012 Dec;12(1):980.
10. Tanimura T, Jaramillo E, Weil D, Raviglione M, Lonnroth K. Financial burden for tuberculosis patients in low- and middle-income countries: a systematic review. Eur Respir J. 2014 Jun 1;43(6):1763–75.
11. Wingfield T, Boccia D, Tovar M, Gavino A, Zevallos K, Montoya R, et al. Defining Catastrophic Costs and Comparing Their Importance for Adverse Tuberculosis Outcome with Multi-Drug Resistance: A Prospective Cohort Study, Peru. PLOS Med. 2014 Jul 15;11(7):e1001675.
12. Wingfield T, Tovar MA, Huff D, Boccia D, Saunders MJ, Datta S, et al. Beyond pills and tests: addressing the social determinants of tuberculosis. Clin Med Lond Engl. 2016;16(6):s79–91.
13. Floyd K, Glaziou P, Houben RMGJ, Sumner T, White RG, Raviglione M. Global tuberculosis targets and milestones set for 2016-2035: Definition and rationale. Int J Tuberc Lung Dis. 2018;22(7):723–30.
14. Laokri S, Dramaix-Wilmet M, Kassa F, Anagonou S, Dujardin B. Assessing the economic burden of illness for tuberculosis patients in Benin: determinants and consequences of catastrophic health expenditures and inequities. Trop Med Int Health TM IH. 2014;19(10):1249–58.
15. Lönnroth K, Glaziou P, Weil D, Floyd K, Uplekar M, Raviglione M. Beyond UHC: Monitoring Health and Social Protection Coverage in the Context of Tuberculosis Care and Prevention. PLoS Med. 2014 Sep 22;11(9):e1001693.
16. Nirmal A, Kuzmik A, Sznajder K, Lengerich E, Fredrick NB, Chen M, et al. 'If not for this support, I would have left the treatment!': Qualitative study exploring the role of social support on medication adherence among pulmonary tuberculosis patients in Western India. Glob Public Health. 2021;0(0):1–13.
17. Lönnroth K, Weil DE. Mass prophylaxis of tuberculosis through social protection. Lancet Infect Dis. 2014;14(11):1032–4.
18. Atkins S, Heimo L, Carter D, Ribas Closa M, Vanleeuw L, Chenciner L, et al. The socioeconomic impact of tuberculosis on children and adolescents: a scoping review and conceptual framework. BMC Public Health. 2022 Nov 23;22(1):2153.

For peer review only - http://bmjopen.bmj.com/site/about/guidelines.xhtml

19. Raišienė AG, Baranauskaitė L. Investigating complexity of intersectoral collaboration: contextual framework for research. Contemp Res Organ Manag Adm. 2018;6(1):79–89.
20. Van Huijstee MM, Francken M, Leroy P. Partnerships for sustainable development: a review of current literature. Environ Sci. 2007;4(2):75–89.
21. Functional Mentoring : A Practical Approach With. 2008;28(3):157–64.
22. Valkokari K, Amitrano CC, Bifulco F, Valjakka T. Collaboration in a Hyperconnected World. Springer Nat. 2016;1:521–30.
23. INE. IV Recenseamento Geral da População e Habitação, 2017 Resultados Definitivos – Moçambique. Inst Nac Estat Maputo-Moçamb. 2019;16–21.
24. World Bank. World Development Indicators [Internet]. 2014. Available from: https://data.worldbank.org/?locations=MZ-XM
25. MISAU. Relatório Anual do Programa. 2021 p. 42.
26. República B da. Lei no 4/2007 de 7 de Fevereiro sobre Protecção social, I série n.6. Lei no 4/2007 de 7 de Fevereiro. 2007.
27. República de Moçambique S de PS. 3º Boletim Estatístico Sobre Protecção Social Ministério. Maputo,; 2020.
28. Benoot C, Hannes K, Bilsen J. The use of purposeful sampling in a qualitative evidence synthesis: A worked example on sexual adjustment to a cancer trajectory. BMC Med Res Methodol. 2016;16(1):1–12.
29. Ness P, Fusch L. Are we there yet? Saturation in qualitative research. Qual Rep. 2015;20(9):1409–16.
30. Graneheim UH, Lundman B. Qualitative content analysis in nursing research: concepts, procedures and measures to achieve trustworthiness. Nurse Educ Today. 2004 Feb;24(2):105–12.
31. Lönnroth K, Jaramillo E, Williams BG, Dye C, Raviglione M. Drivers of tuberculosis epidemics: The role of risk factors and social determinants. Soc Sci Med. 2009 Jun;68(12):2240–6.
32. Lohiniva AL, Mokhtar A, Azer A, Elmoghazy E, Kamal E, Benkirane M, et al. Qualitative interviews with non-national tuberculosis patients in Cairo, Egypt: understanding the financial and social cost of treatment adherence. Health Soc Care Community. 2016;24(6):e164–72.
33. Bongongo T, van der Heever H, Nzaumvila DK, Saidiya CNS. Influence of patients' living conditions on tuberculosis treatment outcomes in a South African health sub-district. South Afr Fam Pract. 2020;62(1):1–6.
34. Lieve Vanleeuw, Zembe-mkabile W, Atkins S. " I ' m suffering for food ": Food insecurity and access to social protection for TB patients and their households in Cape Town , South Africa. 2022;1–16.
35. Union I, Tuberculosis A, Disease L. Public health action. World Health Organ Reg Publ - Eur Ser. 1995;I(56):31–49.
36. Sinha P, Lönnroth K, Bhargava A, Heysell SK, Sarkar S, Salgame P, et al. Food for thought: addressing undernutrition to end tuberculosis. Lancet Infect Dis. 2021;21(10):e318–25.

For peer review only

Legends of the figures

Figure 1: Map showing the study provinces and districts

Figure 2: Structure of Health Care and Basic Social Support System. The black arrow represents the referral from the Social Action Sector, a representation of the National Institute of Social Action (INAS) within the health facility with the responsibility of preparing the documents for support requests, to the Basic Social Support System at INAS

For peer review only

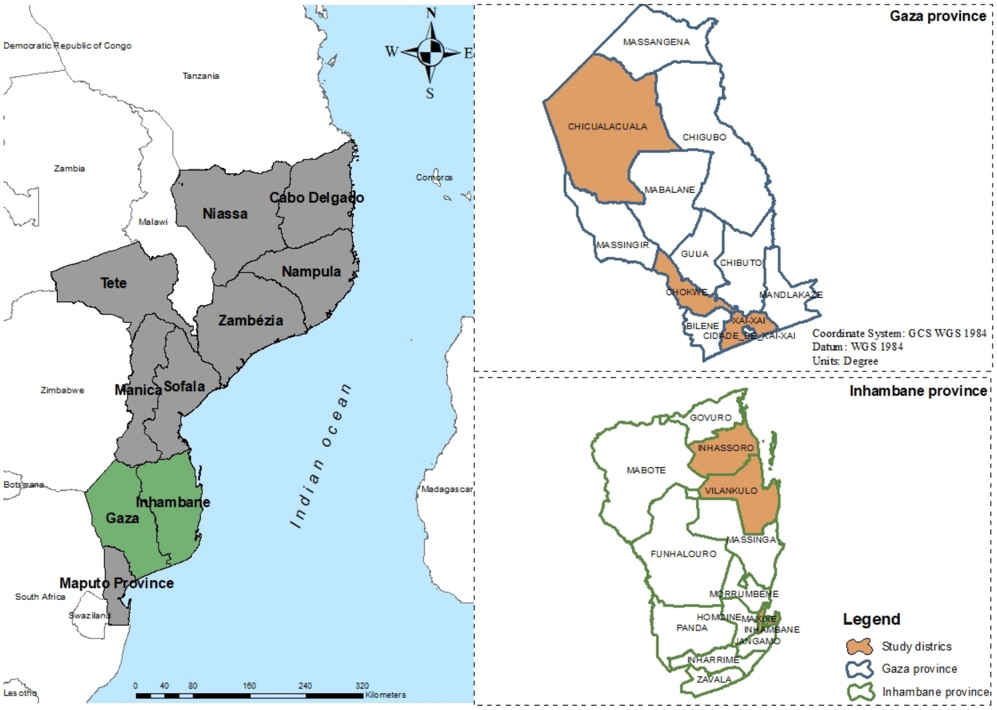

297x210mm (96 x 96 DPI)

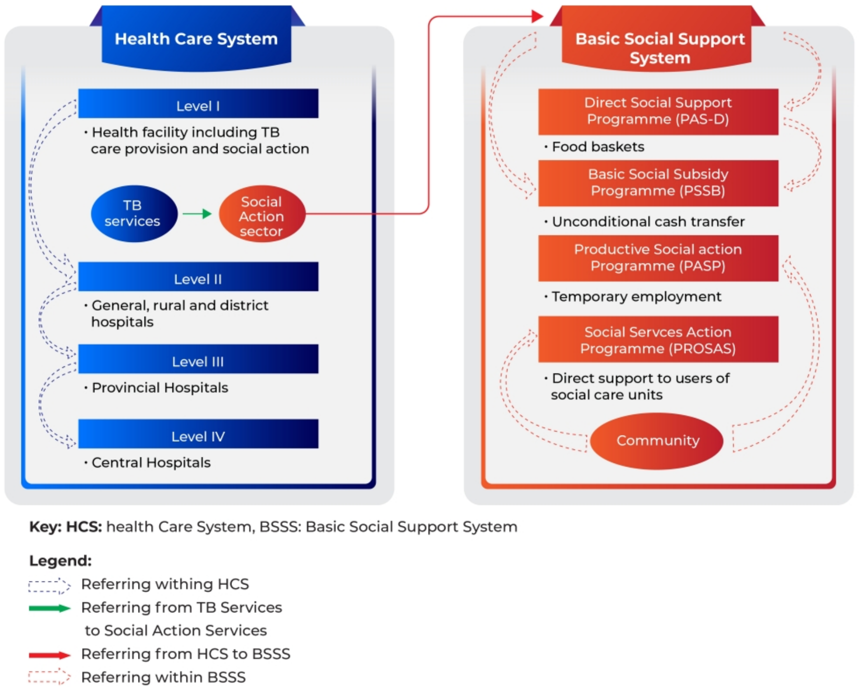

333x245mm (300 x 300 DPI)

SUPPLEMENTARY FILE

Themes	Category	Sub-category	Codes
**(1) Policymakers suggested several solutions but thought that the implementation of many of them would be limited by available resources**	Benefits of supporting people with TB	–	–
	Incurred costs and mitigation	Cost mitigation	Coping mechanisms
			Current mitigation activities
			Provision entity
			Types of strategies for cost mitigation
		Financial impact	Impact on overall economy
			Ability to cope with the TB-related cost
			Impact on the course of disease
			Impact on families' lives
			Impact on patients' lives
		Type of incurred cost	During care seeking
			During treatment
			Factor influencing the occurrence of cost
	Potential solutions for addressing costs	Challenges faced by people with TB	Bottlenecks of TB treatments
			Problems faced by people with TB
			Problems faced by patients' families
		Source of support provision	Availability of support
			Current institutional actions
		Type of needed support	Better housing
			Challenges of receiving direct cash
			Children support / Education for children
			Community actors
			Easy access and expansion of good quality health service
			Home visits through deployment of health workers
			Psychological support
			Cash
			Food
			Transport
**(2) Lack of shared views or processes related to intersectoral collaboration between health and social protection sector hinders design and implementation of social support for people with TB**	Existing collaboration with other sectors	Challenges of collaborations	Barriers
			Facilitators
		Existing collaborations with other sectors	Key stakeholders currently left aside
			Involved stakeholders
			Mechanisms of collaborations and referrals of people with TB
			Type of collaborating institutions

Table 3: Categories and codes created during the coding process on Dedoose software and the themes generated during subsequent analysis

COREQ (COnsolidated criteria for REporting Qualitative research) Checklist

A checklist of items that should be included in reports of qualitative research. You must report the page number in your manuscript where you consider each of the items listed in this checklist. If you have not included this information, either revise your manuscript accordingly before submitting or note N/A.

Topic	Item No.	Guide Questions/Description	Reported on Page No.
**Domain 1: Research team and reflexivity**			
Personal characteristics			
Interviewer/facilitator	1	Which author/s conducted the interview or focus group?	
Credentials	2	What were the researcher's credentials? E.g. PhD, MD	
Occupation	3	What was their occupation at the time of the study?	
Gender	4	Was the researcher male or female?	
Experience and training	5	What experience or training did the researcher have?	
Relationship with participants			
Relationship established	6	Was a relationship established prior to study commencement?	
Participant knowledge of the interviewer	7	What did the participants know about the researcher? e.g. personal goals, reasons for doing the research	
Interviewer characteristics	8	What characteristics were reported about the inter viewer/facilitator? e.g. Bias, assumptions, reasons and interests in the research topic	
**Domain 2: Study design**			
Theoretical framework			
Methodological orientation and Theory	9	What methodological orientation was stated to underpin the study? e.g. grounded theory, discourse analysis, ethnography, phenomenology, content analysis	
Participant selection			
Sampling	10	How were participants selected? e.g. purposive, convenience, consecutive, snowball	
Method of approach	11	How were participants approached? e.g. face-to-face, telephone, mail, email	
Sample size	12	How many participants were in the study?	
Non-participation	13	How many people refused to participate or dropped out? Reasons?	
Setting			
Setting of data collection	14	Where was the data collected? e.g. home, clinic, workplace	
Presence of non-participants	15	Was anyone else present besides the participants and researchers?	
Description of sample	16	What are the important characteristics of the sample? e.g. demographic data, date	
Data collection			
Interview guide	17	Were questions, prompts, guides provided by the authors? Was it pilot tested?	
Repeat interviews	18	Were repeat inter views carried out? If yes, how many?	
Audio/visual recording	19	Did the research use audio or visual recording to collect the data?	
Field notes	20	Were field notes made during and/or after the inter view or focus group?	
Duration	21	What was the duration of the inter views or focus group?	
Data saturation	22	Was data saturation discussed?	
Transcripts returned	23	Were transcripts returned to participants for comment and/or	

For peer review only

Topic	Item No.	Guide Questions/Description	Reported on Page No.
		correction?	
**Domain 3: analysis and findings**			
Data analysis			
Number of data coders	24	How many data coders coded the data?	
Description of the coding tree	25	Did authors provide a description of the coding tree?	
Derivation of themes	26	Were themes identified in advance or derived from the data?	
Software	27	What software, if applicable, was used to manage the data?	
Participant checking	28	Did participants provide feedback on the findings?	
Reporting			
Quotations presented	29	Were participant quotations presented to illustrate the themes/findings? Was each quotation identified? e.g. participant number	
Data and findings consistent	30	Was there consistency between the data presented and the findings?	
Clarity of major themes	31	Were major themes clearly presented in the findings?	
Clarity of minor themes	32	Is there a description of diverse cases or discussion of minor themes?	

Developed from: Tong A, Sainsbury P, Craig J. Consolidated criteria for reporting qualitative research (COREQ): a 32-item checklist for interviews and focus groups. *International Journal for Quality in Health Care*. 2007. Volume 19, Number 6: pp. 349 – 357

Once you have completed this checklist, please save a copy and upload it as part of your submission. DO NOT include this checklist as part of the main manuscript document. It must be uploaded as a separate file.

For peer review only

BMJ Open

Perspectives of healthcare and social support sector policymakers on potential solutions to mitigate financial impact among people with TB in Mozambique: a qualitative study

Journal:	*BMJ Open*
Manuscript ID	bmjopen-2023-073234.R1
Article Type:	Original research
Date Submitted by the Author:	25-Jul-2023
Complete List of Authors:	Nhassengo, Pedroso; Instituto Nacional de Saúde; Karolinska Institute, Department of Global Public Health<br>Yoshino, Clara; Karolinska Institute, Department of Global Public Health; Health and Social Protection Action Research and Knowledge Sharing Network<br>Zandamela, Américo; Instituto Nacional de Saúde<br>De Carmo, Verónica; Instituto Nacional de Saúde<br>Burström, Bo; Karolinska Institutet, Department of Global Public Health<br>Khosa, Celso; Instituto Nacional de Saúde<br>Wingfield, Tom; Liverpool School of Tropical Medicine, Department of International Public Health and Clinical Sciences; Karolinska Institute, Department of Global Public Health<br>Lönnroth, Knut; Karolinska Institute, Department of Global Public Health; Health and Social Protection Action Research and Knowledge Sharing Network<br>Atkins, Salla; Tampere University, New Social Research and Faculty of Social Sciences; Karolinska Institutet, Department of Global Public Health
**Primary Subject Heading**:	Public health
Secondary Subject Heading:	Health policy, Qualitative research
Keywords:	Tuberculosis < INFECTIOUS DISEASES, QUALITATIVE RESEARCH, Public health < INFECTIOUS DISEASES

SCHOLARONE™
Manuscripts

I, the Submitting Author has the right to grant and does grant on behalf of all authors of the Work (as defined in the below author licence), an exclusive licence and/or a non-exclusive licence for contributions from authors who are: i) UK Crown employees; ii) where BMJ has agreed a CC-BY licence shall apply, and/or iii) in accordance with the terms applicable for US Federal Government officers or employees acting as part of their official duties; on a worldwide, perpetual, irrevocable, royalty-free basis to BMJ Publishing Group Ltd ("BMJ") its licensees and where the relevant Journal is co-owned by BMJ to the co-owners of the Journal, to publish the Work in this journal and any other BMJ products and to exploit all rights, as set out in our licence.

The Submitting Author accepts and understands that any supply made under these terms is made by BMJ to the Submitting Author unless you are acting as an employee on behalf of your employer or a postgraduate student of an affiliated institution which is paying any applicable article publishing charge ("APC") for Open Access articles. Where the Submitting Author wishes to make the Work available on an Open Access basis (and intends to pay the relevant APC), the terms of reuse of such Open Access shall be governed by a Creative Commons licence – details of these licences and which Creative Commons licence will apply to this Work are set out in our licence referred to above.

Other than as permitted in any relevant BMJ Author's Self Archiving Policies, I confirm this Work has not been accepted for publication elsewhere, is not being considered for publication elsewhere and does not duplicate material already published. I confirm all authors consent to publication of this Work and authorise the granting of this licence.

For peer review only

Title: Perspectives of healthcare and social support sector policymakers on potential solutions to mitigate financial impact among people with *TB* in Mozambique: a qualitative study

Authors: *Pedroso Nhassengo[1,2,3], Clara Yoshino[2,3], Américo Zandamela[1], Verónica De Carmo[1], Bo Burström[2,3] Celso Khosa[1], Tom Wingfield[2,3,4,5], Knut Lönnroth[2,3], Salla Atkins [2,3,6]*

Affiliations:
[1] Instituto Nacional de Saúde, Marracuene, Mozambique
[2] WHO Collaborating Centre in Tuberculosis and Social Medicine, Department of Global Public Health, Karolinska Institutet, Sweden
[3] Health and Social Protection Action Research and Knowledge Sharing Network (www.sparksnetwork.ki.se), Stockholm, Sweden
[4] Departments of International Public Health and Clinical Sciences, Liverpool School of Tropical Medicine, the UK
[5] Tropical Infectious Diseases Unit, Liverpool University Hospital NHS, Foundation Trust, Liverpool, UK
[6] Health Sciences, Faculty of Social Sciences, Tampere University, Finland

Corresponding Author:
Pedroso Nhassengo
PO box: 264
Email address: pedroso.nhassengo@ins.gov.mz

Word Count: 4983

For peer review only - http://bmjopen.bmj.com/site/about/guidelines.xhtml

ABSTRACT

Objective: People with tuberculosis (TB) and their households face severe socioeconomic consequences, which will only be mitigated by intersectoral collaboration, especially between the health and social sectors. Evidence suggests that key factors for successful collaboration include shared goals, trust, commitment, resource allocation, efficient processes, and effective communication and motivation among collaborating parties. This study aimed to understand healthcare and social support sector policymakers' perspectives on potential solutions to mitigate financial impact among people with TB and their households in Mozambique.

Design: Qualitative study with primary data collection through one-to-one in-depth interviews.

Setting: Gaza and Inhambane provinces, Mozambique.

Participants: Policymakers in health and social support sector.

Results: A total of 27 participants were purposefully sampled. Participants were asked about their perspectives on TB-related financial impact and potential solutions to mitigate such impact. Participants reported that people with TB are not explicitly included in existing social support policies because TB *per se* is not part of the eligibility criteria. People with TB and underweight or HIV were enrolled in social support schemes providing food or cash. Two themes were generated from the analysis: (1) Policymakers suggested several mitigation solutions, including food and monetary support, but perceived that their implementation would be limited by lack of resources; and (2) Lack of shared views or processes related to intersectoral collaboration between health and social support sector hinders design and implementation of social support for people with TB.

Conclusion: Despite health and social sector policymakers reporting a willingness for intersectoral collaboration to mitigate TB-related financial impact, current approaches were perceived to be unilateral. Collaboration between health and social support sectors should focus on improving existing social support programmes.

Strengths and limitations of this study

- In-depth interviews conducted by the first author, who had contextual experience and understanding.
- There was a good representation of participants from both provinces, sectors, gender and age.
- Data collection was conducted in the native language of the first author, ensuring comprehensive understanding and quality control.
- Adaptation of data collection tools based on principles of adaptability, flexibility, and pragmatism.
- This manuscript did not capture the perspectives of people with TB.

For peer review only

INTRODUCTION

Tuberculosis (TB) is one of the leading cause of death due to infection, killing 1.6 million people globally.(1) Until the coronavirus (COVID-19) outbreak, TB was the leading cause of death from a single infectious agent, ranking above HIV/AIDS. Mozambique, which reported 98,000 TB cases in 2021, is on the WHO list of high-TB burden countries that collectively contribute 86% of the global TB burden.(1) The Mozambican health system faces several challenges in addressing this burden including low health provision coverage, low rates of case detection and lack of laboratories for drug susceptibility testing.(1,2) The World Health Organisation's (WHO) 2015 "End TB Strategy" aimed for zero TB-affected households to be facing catastrophic costs (defined as total cost exceeding 20% of annual household income) by the year 2020(3,4) and only 52% of TB-affected households were protected by 2021.(1) This focus on the importance of social support and protecting vulnerable populations was also reinforced in the Sustainable Development Goal 3.3(5) and the United Nations High-Level Meeting on TB (UNHLM-TB).(5,6) In many settings, people with TB incur costs equivalent to more than half of their household's annual income due to TB illness and care seeking.(7,8)

A meta-analysis showed that the proportion of costs among people with TB varies according to the type of TB and co-infection with HIV (TB/HIV). This study further showed that the proportion of people facing catastrophic costs at a cutoff point of 20% of their annual income before TB was 43% among people with Susceptible TB and 80% among people with Drug Resistant TB (DR-TB).(9) Financial impact due to TB and DR-TB has been found to be independently associated with adverse TB treatment outcomes (death during treatment, loss to follow up, treatment failure or recurrent TB disease within 30 months of starting TB treatment).(10,11) People from low socioeconomic strata or those with DR-TB are at a particularly high risk of severe financial impact.(8,10) Therefore, there is a growing consensus that progress towards the "End TB Strategy" targets will require not only investment in strengthening biomedical responses through improving access and effectiveness of healthcare delivery(12) but also actions on the socio-economic determinants and consequences of TB, as well as measures to improve quality of life and wellbeing after TB-episode(3,4,10,13), especially in low- and middle-income Countries (LMIC). The holistic impact of TB on wellbeing, finances, and quality of life requires the simultaneous provision of biomedical and socioeconomic approaches to control TB, which in turn requires intersectoral collaboration especially between healthcare and social sectors.(14–16) However, despite a high proportion of people with TB and their households being economically vulnerable and likely to benefit significantly from social support, they are rarely covered by social support policies.

In Mozambique as in many other settings, TB disease is not a formal criterion for social support, despite its severe consequences for people and families affected by it.(15) To begin developing potential for such support requires collaboration between different sectors. To date, however, there is little knowledge about how a collaboration or pathway between the healthcare and social support sector would, or could, work in practice in Mozambique. Our study therefore focuses on understanding the current landscape of collaboration, and the potential for future collaboration. To the extent of our knowledge, financial impact faced by people with TB and their households and the role of intersectoral collaborations to address such impact has not previously been studied in Mozambique.

For peer review only - http://bmjopen.bmj.com/site/about/guidelines.xhtml

We aimed to understand healthcare and social support sector policymakers' perspectives on potential solutions to mitigate financial impact among people with TB and their households in Mozambique, a low-income high TB burden country.

METHODS

Conceptual framework

The theory underlying this study is that although intersectoral collaboration is complex and sometimes hard to achieve, it will be one of the fundamental issues to consider and address in order to mitigate financial impact of TB-affected households. Common goals, mutual trust and commitment to share the risks, allocation of time and resources are unquestionable preconditions for a sustainable collaboration. Other dimensions to be accounted for include the scope and type of collaboration, efficiency of the collaboration processes and management of the activities and interaction between collaborating parties, which encompasses communication and motivation to collaborate.(16–18)

Study design

This was a qualitative phenomenological study with primary data collection using one-to-one in-depth interviews with policymakers either in healthcare or in social support sectors. This study was part of the CHEST project implemented in Mozambique, whose main objective was to estimate the prevalence of people incurring catastrophic costs due to TB and develop a bi-directional referral system between health and social services to increase access of grants by people with TB.

Involvement of patients and general public

Patients or the public were not involved in the design, conduct, reporting, or dissemination plans of our research.

Setting

Mozambique is a Sub-Saharan African country with an estimated population of 31 million people(19), a literacy rate of 61%, and 63.7% living on less than $1.90 a day.(20) Study participants were recruited in seven districts of Inhambane and Gaza provinces in southern Mozambique (Figure 1). These provinces had 28 districts that jointly contributed to 12.8% of the country's TB notifications in 2021.(21) Since all 28 districts met the study site inclusion criteria, we randomly selected two districts in each province considering the type of districts and geographical representation and, purposely included the provincial representations of both sectors as they present distinct socioeconomic landscapes and Vilanculos as it shared social sector representation with Inhassoro (a selected district). All other selected districts had both health and social sector representation.

For peer review only - http://bmjopen.bmj.com/site/about/guidelines.xhtml

Structure of healthcare and social protection system

The current Mozambican public healthcare services encompass four levels (figure 2). The lower level provides primary healthcare including free-of-charge TB services (diagnosis and treatment)(2) and initial assessment for social support through the Social Action Sector. The Social Support services are delivered by the National Institute of Social Action (INAS) following four operational programmes namely: **i) Basic Social Subsidy Programme (PSSB)** providing regular unconditional cash transfer to vulnerable people (mainly people older than sixty and people with disabilities); **ii) Direct Social Support Programme (PAS-D)** providing food baskets to vulnerable people and households including people with chronic illnesses; **iii) Productive Social Action Programme (PASP)** providing temporary employment in urban and rural areas and **iv) Social Services Action Programme (PROSAS)** providing direct support to users of social care units that provides daily meals to elderly people, homeless and orphan in specific places.(22,23) There is no social support scheme available specifically targeted towards TB-affected people and their households. Both healthcare and social support sectors have paper-based registration.

Study participants and data collection

The participants were purposefully recruited in the selected districts between October 2021 and February 2022 using snowballing and maximum variation sampling.(24) The research team compiled a list of work positions of interest in both sectors. Pre-recruitment was conducted either by email or phone. A total of 34 people were invited to participate, however, 7 were not able to participate due to personal reasons and two of those were replaced by an equivalent respondent. Eligibility criteria were (1) age 18 years or older, (2) with at least one year of active work in the current position and (3) capable of providing informed consent. Informed consent and data were collected face-to-face by the first author (PN). Interviews were conducted in a quiet and private place at the participant's offices and lasted between 30 to 90 minutes. We stopped data collection with 27 participants when we reached saturation.(25) Interviews were conducted following a semi-structured topic guide that covered three major sections: i) perceptions of financial impact faced by people with TB; ii) potential solutions to mitigate the financial impact; and iii) barriers and facilitators for intersectoral collaboration (Supplementary file 1). Interviews were audio-recorded, transcribed *verbatim* and translated from Portuguese into English. Transcriptions were controlled for quality against the recordings by Research Assistants (RAs) and against translations by PN. Non-verbal communication and other important information were captured as field notes during each interview.

Of a total of 27 participants enrolled, 14 (52%) were from Gaza and 13 (48%) were from Inhambane province. The overall median age was 37 (Interquartile Range, IQR: 32 - 47) years old. Most respondents were male (56%) and the median time at the working position was four years (IQR: 3 – 5). About three quarters of the participants had obtained a university degree (Table 1).

For peer review only - http://bmjopen.bmj.com/site/about/guidelines.xhtml

Demographic Characteristics	Gaza n=13	Inhambane n=14	Total n=27
**Median Age (IQR)**	**33 (32-45)**	**39 (33-48)**	**37 (32-47)**
**Gender n(%)**			
Male	7 (53.9)	8 (57.1)	15 (55.6)
Female	6 (46.2)	6 (42.7)	12 (44.4)
**Work Position n(%)**			
Healthcare policymakers	**8 (61.5)**	**8 (57.1)**	**16 (59.3)**
District/clinical directors	3 (23.1)	2 (14.3)	5 (18.5)
Public health/TB focal points	5 (38.5)	6 (42.9)	11 (40.7)
Social Support policymakers	**5 (38.5)**	**6 (42.9)**	**11 (40.8)**
Programme managers	0 (0)	2 (14.3)	2 (7.4)
Social support focal points	4 (30.8)	3 (21.4)	7 (25.9)
District directors	1 (7.7)	1 (7.1)	2 (7.4)
**Median Time in Work Position in years (IQR)**	**4 (3-5)**	**3.5 (2-4)**	**4 (2-5)**
**Education n(%)**			
Less than Secondary School	0 (0)	0 (0)	0 (0)
Secondary School	5 (38.5)	3 (21.4)	8 (29.6)
University	8 (61.5)	11 (78.6)	19 (70.4)

Table 1. Participants' demographic characteristics

Data Analysis

Qualitative content analysis was adopted as proposed by Graneheim and Lundman.(26) Data were deductive and inductively coded by PN and CY using Dedoose software and analysed by PN, CY and SA. Three interviews were double coded to verify the similarity of the encodings. For independent coding, the researchers had regular discussions about the understanding of the codes. The deductive coding was based on the topic guide and preliminary analysis of the interviews. This was followed by inductive coding with the inclusion of codes that emerged from the data during the deductive coding. The interview text was sorted into four content areas: i) incurred cost and mitigation; ii) types of support for people with TB; iii) benefits of supporting people with TB and iv) intersectoral collaboration. Texts corresponding to these areas were extracted from the interview transcriptions and brought together into one text, which formed the unit of analysis. The text was divided into meaning units that were condensed. The condensed meaning units were, abstracted and labelled with a code. Through a manifest analysis, relevant codes to the research questions were rearranged and grouped into categories (Table 2). The last stage consisted of interpreting the data through a latent analysis and developing the themes. Full coding process is presented in the Supplementary File 2.

For peer review only - http://bmjopen.bmj.com/site/about/guidelines.xhtml

Theme	Policymakers suggested several mitigation solutions, including food and monetary support, but perceived that their implementation would be limited by lack of resources			Lack of shared views or processes related to intersectoral collaboration between health and social protection sector hinders design and implementation of social support for people with TB		
Category	Potential solutions for addressing costs			Existing collaboration with other sectors		
Codes	Food, Cash and transport	Mobile brigades and expansion of quality healthcare system	Better housing and psychological support	Mechanisms of collaboration	Involved stakeholders	Type of collaborating institutions

Table 2. Example of coding

Researcher characteristics and reflexivity

The Interviews were conducted by PN. He is a medical doctor and PhD candidate with seven-years of experience in qualitative research. PN's research is based in Mozambique, and he is familiar with the health and social services system. Coding was conducted by PN and CY, who are both native Portuguese speakers and fluent English speakers. She is a social scientist and public policy researcher. CY and SA have experience in qualitative and international social protection systems.

PN, being affiliated with a central public institution, recognized that conducting research at the provincial level could influence participants to provide favourable opinions. Therefore, PN invested significant effort in explaining study procedures, obtaining informed consent, and addressing participants' concerns. The interview began only after establishing rapport and trust between PN and the participants.

CY's experiences of similar health and social services from Brazil added value to the analysis. She immersed herself in the data and got a thorough understanding of its the content and context. The team's experience on TB and social protection in Africa and globally improved the understanding of the research findings.

Ethical considerations

All participants were provided detailed information about the study and provided a written informed consent before the interview**.** The study was performed in accordance with the study protocol, the Declaration of Helsinki (last update: October 2013), was approved by the Institutional Ethics Committee of the *Instituto Nacional de Saúde* in Maputo, Mozambique (Ref. no. 001/CIBS-INS/2020) and by the Swedish Ethical Review Authority in Stockholm, Sweden (Ref. no. 2022-03297-01). Interviews were anonymized to protect participant's identity.

For peer review only

RESULTS

Perceptions of financial impact faced by people with TB and its consequences

Healthcare and social sector policymakers had different understanding of the need and provision of support for people with TB. Social sector's policymakers had little knowledge of the treatment and care pathways of people with TB and thus their need for support. On the other hand, healthcare policymakers were aware of the impact of TB on people's lives, and the socioeconomic implications of treatment. They reported that TB-related costs were high for people with TB and their households, and this was worse when the Primary Income Earner (defined as the person with the largest economic contribution to secure the family's basic needs) was affected or the person had DR-TB. Incurred costs due to transportation, food and income loss seemed to be the major causes of negative financial impact of TB.

Theme 1: Policymakers suggested several mitigation solutions, including food and monetary support, but perceived that their implementation would be limited by lack of resources

Current availability and source of social support provision

The current availability of support for people with TB was seen as inadequate by both healthcare and social sector policymakers. While institutional actions in the healthcare sector included active tracing by community workers and decentralization of TB services to remote areas, social protection sector provided food and monetary support through PAS-D and PSSB. Beyond TB services related measures, healthcare sector could not recount other support. Both sectors mentioned the potential benefit of psychological support to people with TB and their families focusing on the disease, treatment of TB and, enrichment of food through practical mentoring about their diet. This was perceived as having positive effects including decreasing the need for support and demand for supplements at health facilities.

Community centred strategies and expansion of TB Services

Healthcare policymakers described extensively the community-based approaches that they perceived to reduce direct and indirect out-of-pocket medical costs. They mentioned, for example, brigades, which are a group of healthcare workers whose responsibilities can include community contact tracing, community based Directly Observed Therapy (DOTc), TB screening and sample collection, home visits and follow-up of people under treatment. Community-based strategies were seen as key to people with TB mainly due to reduction of travelling costs.

> *"One of our ways to reduce the cost [of TB treatment] is the community DOT where we have our activists there. [...] this way we reduce the cost of [people with TB] who have to come to the health facility twice a month." (Male, healthcare policymaker, 3 years in position)*

For peer review only - http://bmjopen.bmj.com/site/about/guidelines.xhtml

Expansion of healthcare services to more remote areas was considered by healthcare policymakers as a suitable strategy to reduce transportation cost and waiting time at the health facilities. They reported the expansion of TB treatment sites having as example the antiretroviral therapy (ART) services, which was expanded to all health facilities and resulted in perceived reduction of transportation cost. They also suggested that counsellors (staff dedicated to counselling at health facilities or at communities) and health technicians (staff with basic medical training and abilities to diagnose and prescribe medications for the most common diseases) should work together as community health workers. The current approach for community work was seen as inappropriate as the healthcare sector was mainly represented by counsellors who lack clinical knowledge. On the other hand, health technicians or community health workers were perceived to be cheaper than medical doctors.

Food provision and monetary support for people with TB

According to both healthcare and social sector policymakers, the existing policy on provision of social support for people with TB was conditional on having other conditions such as HIV and underweight and, they were eligible to receive food supplements, as part of a collaboration with the nutrition programme.

From the social sector's side, people on ART or chronically ill (illnesses requiring long-term care and support) received a basic food basket containing nutritional supplementation through the Direct Social Support Programme (PAS-D). The programme initially covers six months of continuous support after which recipients are re-assessed regarding the need for continued support. If so, the support could be extended for additional six months or the recipient would be redirected to the Basic Social Subsidy Programme (PSSB), which offers lifelong monetary support. However, having TB was not *per se* an eligibility criterion for enrolling in this programme.

One social sector policymaker (Female, social sector policymaker, 3 years in position) reported that people with chronic illnesses used to receive food baskets, but due to lack of funds, they started to receive cash, as they have a lower cost compared to PAS-D food baskets. They also mentioned that there is a direct cash support for transport. However, people with TB face difficulties in receiving such support, as it requires them to provide proof of transport payment, which is seldom available on public transportation.

Barriers and facilitators of addressing TB-related costs

Participants reported different strategies undertaken by both institutions and households to mitigate TB-related financial impact. Healthcare policymakers reported strategies such as obtaining additional income by selling livestock and spouses whose husband with TB was primary income earner taking over financial responsibilities.

For peer review only - http://bmjopen.bmj.com/site/about/guidelines.xhtml

Cost mitigation activities conducted by both policymakers included institutionalised and non-institutionalised actions. Institutionalised actions were defined as activities implemented according to the operational procedures and non- institutionalised actions were defined as activities or actions implemented by policymakers' instant initiative. The latter resulted from participants being sensitised by the situation of the person with TB and helping them using their own resources. These consisted of different levels of involvement, from healthcare policymakers advising people with TB on possible ways to obtain some income to mobilising the community to donate food or money. Social sectors' policymakers also reported trying to enrol people with TB in any social protection programme.

Strategies to mitigate TB-related financial impact were reported by healthcare policymakers as still insufficient, as people with TB continue to incur high costs and therefore risk to abandon the treatment.

Potential solutions for addressing TB-related costs

Both healthcare and social sector policymakers thought that food was the most important social support option for people with TB, because the need for food cannot be compensated through other means, and many people with TB were food insecure. Lack of transport, for example, could be replaced by walking in the absence of cash; but food is a staple that is always needed by people with TB. Healthcare policymakers also reported that food was the most common form of support that people with TB asked for. However, food management was considered complicated, as it requires more complex logistics (purchase, storage, and distribution).

> *"I would prioritise food because we end up having other alternatives for transport but for food, we don't have any measure that helps us to minimise the situation of [people with TB]" (Male, healthcare policymaker, 6 years in position).*

According to policymakers, the lack of food could be countered either by direct provision of food or by provision of cash. However, healthcare policymakers were reluctant to give cash due to the perceived risk of misuse, such as alcohol consumption. Despite this, social sector reported that cash provision would be practically and logistically easier and that most people would adequately use the cash, as they were instructed during psychological support. Both healthcare and social sector policymakers seemed to believe that addressing only other financial burdens, such as transportation cost, is not enough if food was not provided.

> *"[...] money, as such, I don't think so. Money creates problems ahead, that's why I am not suggesting money support. A basic food basket is better because when we give money to certain people who are undergoing treatment when they start to improve instead of buying food, they deviate the money." (Male, healthcare policymaker, 3 years in position).*

For peer review only - http://bmjopen.bmj.com/site/about/guidelines.xhtml

Theme 2: Lack of shared views or processes related to intersectoral collaboration between health and social protection sector hinders design and implementation of social support for people with TB.

Barriers and facilitators to collaboration between healthcare and social sectors

Participants perceived involvement of key stakeholders as an important step towards appropriate cost-mitigation strategies. The Ministry of Health, through its provincial and district representations and the Ministry of Gender, Children and Social Action, through INAS and its provincial and district representations are considered the most important stakeholders. Other governmental stakeholders played complementary roles. The community and churches providing informal social support, were also considered important players. Participants also suggested that all stakeholders working in similar areas or in philanthropic projects should be engaged to strengthen the collaborations.

Support for people with disabilities or chronic illness was predominant in the collaboration between the healthcare and social institutions. Both healthcare and social sector participants mentioned that there was an ongoing intersectoral collaboration for supporting people with chronic illnesses. A substantial barrier was seen as the non-inclusion of TB disease in Mozambican social support policies which prevented direct provision of social support to people with TB. An alternative measure was registering people with TB according to other chronic illness or disability that they might have such as chronic lung disease or malnutrition. Overall, healthcare policymakers seemed dissatisfied with the current collaboration. Waiting time for support to be provided, poor communication, perceived lack of resources and cumbersome vulnerability assessment were reported as being the source of dissatisfaction. They described waiting time for support provision as long and inadequate for people with TB and opined that often the social sector counterpart delayed the feedback of support applications.

> *"The most obvious barrier is the delay in response [...] because the feedback of the applications take time. At some point, we get the response while the patient has already finished the treatment or maybe others have already defaulted or died." (Female, healthcare policymaker, 33 years in position).*

Participants from the healthcare sector seemed to not understand how the social sector proceeds for support allocation. Information about the correct steps as well as the eligibility for support provision seemed to be scarce. On the other hand, social sector assists very few people with chronic illness (including TB), compared to elderly people and children. This may be explained by limited dissemination of the programmes, lack of understanding and low referral rates by the health sector. Additionally, both participants reported resources limitation to cover new applications. Allocated budget and goals were pre-defined at central level and new beneficiaries entered the programmes only when current beneficiaries were discharged from it resulting in long waiting list.

On top of this, the lifelong support provided to beneficiaries with chronic illnesses was perceived to be a small amount varying according to the number of household members (from $8 to 16 per month). Almost all participants agreed that the amount was insufficient to handle basic needs even for a single-person household.

Participants from social sector stressed that the sector, understandably, lacks clinical knowledge or competence. Participants reported that a health technician or a psychologist allocated to their offices would improve the identification of sick beneficiaries at the community level and promptly refer them to the healthcare sector for further management as appropriate.

Intersectoral communication was raised as an area for improvement by healthcare sector but not social sector participants. One healthcare participant suggested that the intersectoral communication could be more informal and formalities could be left to a later stage and that high-level intersectoral policymaking meetings and discussions could be of benefit to define common goals and consider how best intersectoral collaboration could successfully be implemented and achieved.

Mechanisms of collaboration between healthcare and social sectors

The roles of the two major players in supporting people with TB seemed clear to all participants. The health sector's responsibility was to diagnose the TB disease, check clinical eligibility and pre-assessment of vulnerability, which enabled them to refer these people to the Social Action Sector. On the other hand, the Social Action Sector should do a more complete and detailed vulnerability assessment and submitting of the application to social sector at INAS. The expectations were that INAS would provide approval for enrolment and support provision if the budget was available. Another direction of collaboration was related to how INAS' beneficiaries reach the health sector when they become sick (people with presumptive TB for example). This was unclear and may not be in place as INAS' lack of competence to assess health status limited them to counselling and advising people to go to the health facilities for better assessment and diagnosis.

DISCUSSION

This study revealed healthcare and social sector policymakers' perceptions of the financial impact and challenges faced by people with TB in Mozambique as well as current and potential joint mitigation strategies between the two sectors. Since TB is not considered an eligibility criterion in current Mozambican social support policies, people with TB and their households are not covered by adequate and equitable social support. Our findings provide valuable information about both the current provision of social support and gaps in support for people with TB, suggested solutions for support provision during treatment phase and dynamics of intersectoral collaboration between healthcare and social support sector.

For peer review only - http://bmjopen.bmj.com/site/about/guidelines.xhtml

People from low socioeconomic strata are disproportionately affected by TB, and our interviewees were clear, at least in the healthcare sector, that social determinants are the drivers of TB in Mozambique. Similar to other studies, there were vulnerabilities among people with TB that included poverty, low income, food insecurity, undernutrition(27) and risk of income loss. This was due to their livelihoods being based predominantly on farming, self-employment or informal work, with many unable to benefit from stable labour activities(28), and health and sickness insurance coverage. This financial situation meant an inability to afford the costs associated with TB. Studies in Africa and Asia also reported similar income and job-related problems.(29) Distance to health facilities and its related transportation costs were perceived as negatively impacting health seeking behaviour, leading to delays in presenting at health facilities, worsening of health condition and risk of spreading the disease to cohabiting people.(7) Health system-related delays in diagnosis also contribute to increasing the financial impact due to costs with transportation and out-of-pocket payments of medication taken during waiting time. We also found that healthcare policymakers thought that the TB-related costs and consequences were exacerbated by rigid DOT routines. Most people with TB struggle to have enough food and money for daily transportation to DOT sites. Distance and transportation cost to the DOT site, costs of food, out-of-pocket medication and income loss were the leading causes of high costs during treatment and lack of transportation money during TB treatment was associated with missing DOT visits and TB doses and lack of food with treatment failures.(8)

Although lack of food and money negatively impacted the TB treatment, food provision was the most important due to its impact on treatment adherence and medication intake. Similar studies showed that food support was the most important for more easily averting the challenges faced by people with TB. However, our findings indicate that food provision requires a more complex logistical apparatus for purchasing, storage, and distribution to beneficiaries making the cash preferred over food support and cash based schemes are less costly than food-based interventions.(30) The impact of existing cash support schemes was reported to be limited by low cash amounts provided. A systematic review by Todd et al. discussed the consensus that that the cash transferred value should be large enough to mitigate the TB-related financial impact and motivate households to remain in care, while being too small to potentially act as a coercion.(31) Psychological support currently provided by healthcare and social support sectors to people with TB and their household members was considered indispensable, but insufficient by itself. A study by Ahuja et al. in western India(32) showed that family support to people with TB improved their ability to remain in care. However, South African evidence suggests that while family can be an important support structure, as their resources are limited, people with TB are left without important sources of support.

Ultimately, existing collaborations between the two key players could improve referral and support for people with TB and their households. However, lack of local evidence of disease related financial and social impact might hamper better understanding of the need for urgent intervention and collaboration. Alignment of aims and goals between the healthcare and social support sector was likely to have been influenced by lack of official collaboration

For peer review only - http://bmjopen.bmj.com/site/about/guidelines.xhtml

establishment, definition of roles of the parties, specific action plan to be carried out, benefits and necessary resources.(16) Collaboration should be stablished and regulated at central level ensuring definition of goals and roles of the parties. Local level institutions should adapt the collaboration to fit the available resources and needs of the affected people.

In light of this, locally based research to assess the TB related financial and social impact is required to support the inclusion of people with TB in the currently available social support schemes and improve the collaboration and actions at the policy-making level.

STRENGTHS AND LIMITATIONS

Strengths of this study included good representation of policymakers both in terms of provinces, sectors, gender and age, high quality and depth of interviews, and data collection conducted by PN. This allowed a comprehensive understanding of the responses and quality control of the transcripts in Portuguese and its translation to English. Additionally, data collection tools were adapted during the process following the principles of adaptability, flexibility, and pragmatism. An important limitation of this study was the timing of the data collection, which coincided with the period when state servants usually go for annual leave. Because of this, some potential study participants could not be reached. In this situation, the interview was made with the person responding to the position at the time of data collection. This manuscript did not capture the perspectives of people with TB, an important information to triangulate our data. Despite this, our findings are consistent with existing literature discussing solutions of TB-related costs.

CONCLUSIONS

TB disease is still perceived as a disease of poverty, driven by social determinants and with severe socioeconomic consequences. Our findings from key health and social sector policy makers suggest that the two sectors are willing to collaborate beyond the current modest and unilateral efforts to mitigate the financial hardship of TB. Collaboration across sectors should be strengthened and eligibility criteria for social support should be revised to explicitly include people with TB to enable mitigation of costs of care and compensation of foregone income. Lack of financial and human resources hampers implementation of several identified strategies that could potentially avert the TB-related cost faced by people with TB and their households. Further research is needed to explore reasons of non-inclusion of TB patients as eligible beneficiaries, as well as barriers for broader policy changes for social protection inclusion of people with TB.

ACKNOWLEDGMENTS

The authors express their gratitude to the Ministry of Health and the Ministry of Gender, Children and Social Action from Mozambique and study participants for their participation and support. We also would like to thank the Mozambican National Tuberculosis Programme for

For peer review only

its contribution to setting up the study. Lastly, we would like to thank Dr Kerry Viney for her support in designing the study and reviewing the paper.

CONTRIBUTORSHIP STATEMENT

Fund acquisition and study design: Salla Atkins, Pedroso Nhassengo, Celso Khosa, Tom Wingfield, Bo Burström and Knut Lonnröth
Data collection: Pedroso Nhassengo, Clara Yoshino, Américo Zandamela, Verónica De Carmo, Bo Burström, Celso Khosa, Tom Wingfield, Knut Lönnroth, Salla Atkins
Data analysis: Pedroso Nhassengo, Clara Yoshino, Américo Zandamela, Verónica De Carmo and Salla Atkins
Writing of the first draft: Pedroso Nhassengo, Clara Yoshino, Américo Zandamela, Verónica De Carmo and Salla Atkins
Review and editing of the manuscript: Pedroso Nhassengo, Clara Yoshino, Américo Zandamela, Verónica De Carmo, Bo Burström, Celso Khosa, Tom Wingfield, Knut Lönnroth, Salla Atkins
All authors read and approved the final version of the manuscript.

COMPETING INTERESTS

No competing interest.

FUNDING STATEMENT

This study was funded by Vetenskapsrådet through the Swedish Research Council, VR 2017-05497. TW is supported by grants from the Wellcome Trust, UK (209075/Z/17/Z), the Medical Research Council, Department for International Development, and Wellcome Trust (Joint Global Health Trials, MR/V004832/1), and a Dorothy Temple Cross Tuberculosis International Collaboration Grant from the Medical Research Foundation (MRF-131-0006-RG-KHOS-C0942), UK.

DATA SHARING STATEMENT

No additional data available.

For peer review only

REFERENCES

1. WHO. Global tuberculosis report 2022 [Internet]. Geneva; 2022 [cited 2023 May 22]. Available from: https://www.who.int/teams/global-tuberculosis-programme/tb-reports/global-tuberculosis-report-2022
2. Saúde M da. Avaliação e manejo de patientes com TB, Normas Nacionais, Maputo. 2019.
3. World Health Organization. Implementing the End TB Strategy: The Essentials. 2015;1–130.
4. WHO. Implementing the end TB strategy the essentials, 2022 update [Internet]. 2022 [cited 2023 Mar 7]. Available from: https://www.who.int/publications/m/item/implementing-the-end-tb-strategy-the-essentials-2022-update
5. United Nations. UN General Assembly, Transforming our world : the 2030 Agenda for Sustainable Development, 21 October 2015, A/RES/70/1, available at: https://www.refworld.org/docid/57b6e3e44.html [accessed 28 November 2019]. 2015;16301(October):1–35.
6. Stop TB Partnership. UNHLM on TB: key targets and commitments. Geneva, Switzerland: STOP TB Partnership; 2020.
7. Barter DM, Agboola SO, Murray MB, Bärnighausen T. Tuberculosis and poverty: the contribution of patient costs in sub-Saharan Africa – a systematic review. BMC Public Health. 2012 Dec;12(1):980.
8. Tanimura T, Jaramillo E, Weil D, Raviglione M, Lonnroth K. Financial burden for tuberculosis patients in low- and middle-income countries: a systematic review. Eur Respir J. 2014 Jun 1;43(6):1763–75.
9. Ghazy RM, Saeh HME, Abdulaziz S, Hammouda EA, Elzorkany A, Kheder H, et al. A Systematic Review and Meta-Analysis on Catastrophic Cost incurred by Tuberculosis Patients and their Households. medRxiv. 2021 Mar 1;2021.02.27.21252453.
10. Wingfield T, Boccia D, Tovar M, Gavino A, Zevallos K, Montoya R, et al. Defining Catastrophic Costs and Comparing Their Importance for Adverse Tuberculosis Outcome with Multi-Drug Resistance: A Prospective Cohort Study, Peru. PLOS Med. 2014 Jul 15;11(7):e1001675.
11. Wingfield T, Tovar MA, Huff D, Boccia D, Saunders MJ, Datta S, et al. Beyond pills and tests: addressing the social determinants of tuberculosis. Clin Med Lond Engl. 2016;16(6):s79–91.
12. Floyd K, Glaziou P, Houben RMGJ, Sumner T, White RG, Raviglione M. Global tuberculosis targets and milestones set for 2016-2035: Definition and rationale. Int J Tuberc Lung Dis. 2018;22(7):723–30.
13. Laokri S, Dramaix-Wilmet M, Kassa F, Anagonou S, Dujardin B. Assessing the economic burden of illness for tuberculosis patients in Benin: determinants and consequences of catastrophic health expenditures and inequities. Trop Med Int Health. 2014 Oct;19(10):1249–58.
14. Lönnroth K, Weil DE. Mass prophylaxis of tuberculosis through social protection. Lancet Infect Dis. 2014;14(11):1032–4.
15. Atkins S, Heimo L, Carter D, Ribas Closa M, Vanleeuw L, Chenciner L, et al. The socioeconomic impact of tuberculosis on children and adolescents: a scoping review and conceptual framework. BMC Public Health. 2022 Nov 23;22(1):2153.
16. Raišienė AG, Baranauskaitė L. Investigating complexity of intersectoral collaboration: contextual framework for research. Contemp Res Organ Manag Adm. 2018;6(1):79–89.
17. Van Huijstee MM, Francken M, Leroy P. Partnerships for sustainable development: a review of current literature. Environ Sci. 2007;4(2):75–89.

For peer review only - http://bmjopen.bmj.com/site/about/guidelines.xhtml

18. Functional Mentoring : A Practical Approach With. 2008;28(3):157–64.
19. INE. IV Recenseamento Geral da População e Habitação, 2017 Resultados Definitivos – Moçambique. Inst Nac Estat Maputo-Moçamb. 2019;16–21.
20. World Bank. World Development Indicators [Internet]. 2014. Available from: https://data.worldbank.org/?locations=MZ-XM
21. MISAU. Relatório Anual do Programa [Internet]. 2022 [cited 2022 May 9] p. 42. Available from: https://www.misau.gov.mz/
22. Boletim da República. Lei nº 4/2007 de 7 de Fevereiro sobre Protecção social. 2007;I série, n.
23. República de Moçambique S de PS. 3º Boletim Estatístico Sobre Protecção Social Ministério. Maputo,; 2020.
24. Benoot C, Hannes K, Bilsen J. The use of purposeful sampling in a qualitative evidence synthesis: A worked example on sexual adjustment to a cancer trajectory. BMC Med Res Methodol. 2016;16(1):1–12.
25. Ness P, Fusch L. Are we there yet? Saturation in qualitative research. Qual Rep. 2015;20(9):1409–16.
26. Graneheim UH, Lundman B. Qualitative content analysis in nursing research: concepts, procedures and measures to achieve trustworthiness. Nurse Educ Today. 2004 Feb;24(2):105–12.
27. Lönnroth K, Jaramillo E, Williams BG, Dye C, Raviglione M. Drivers of tuberculosis epidemics: The role of risk factors and social determinants. Soc Sci Med. 2009 Jun;68(12):2240–6.
28. Lohiniva AL, Mokhtar A, Azer A, Elmoghazy E, Kamal E, Benkirane M, et al. Qualitative interviews with non-national tuberculosis patients in Cairo, Egypt: understanding the financial and social cost of treatment adherence. Health Soc Care Community. 2016;24(6):e164–72.
29. Bongongo T, van der Heever H, Nzaumvila DK, Saidiya CNS. Influence of patients' living conditions on tuberculosis treatment outcomes in a South African health sub-district. South Afr Fam Pract. 2020;62(1):1–6.
30. Lieve Vanleeuw, Zembe-mkabile W, Atkins S. " I ' m suffering for food ": Food insecurity and access to social protection for TB patients and their households in Cape Town , South Africa. 2022;1–16.
31. Todd H, Hudson M, Grolmusova N, Kazibwe J, Pearman J, Skender K, et al. Social Protection Interventions for TB-Affected Households: A Scoping Review. Am J Trop Med Hyg. 2023 Apr;108(4):650–9.
32. Nirmal A, Kuzmik A, Sznajder K, Lengerich E, Fredrick NB, Chen M, et al. 'If not for this support, I would have left the treatment!': Qualitative study exploring the role of social support on medication adherence among pulmonary tuberculosis patients in Western India. Glob Public Health. 2021;0(0):1–13.

For peer review only

Legends of the figures

Figure 1: Map showing the study provinces and districts.

Figure 2: Current structure of Healthcare and Basic Social Support System. The black arrow represents the referral from the Social Action Sector, a representation of the National Institute of Social Action (INAS) within the health facility with the responsibility of preparing the documents for support requests, to the Basic Social Support System at INAS.

For peer review only

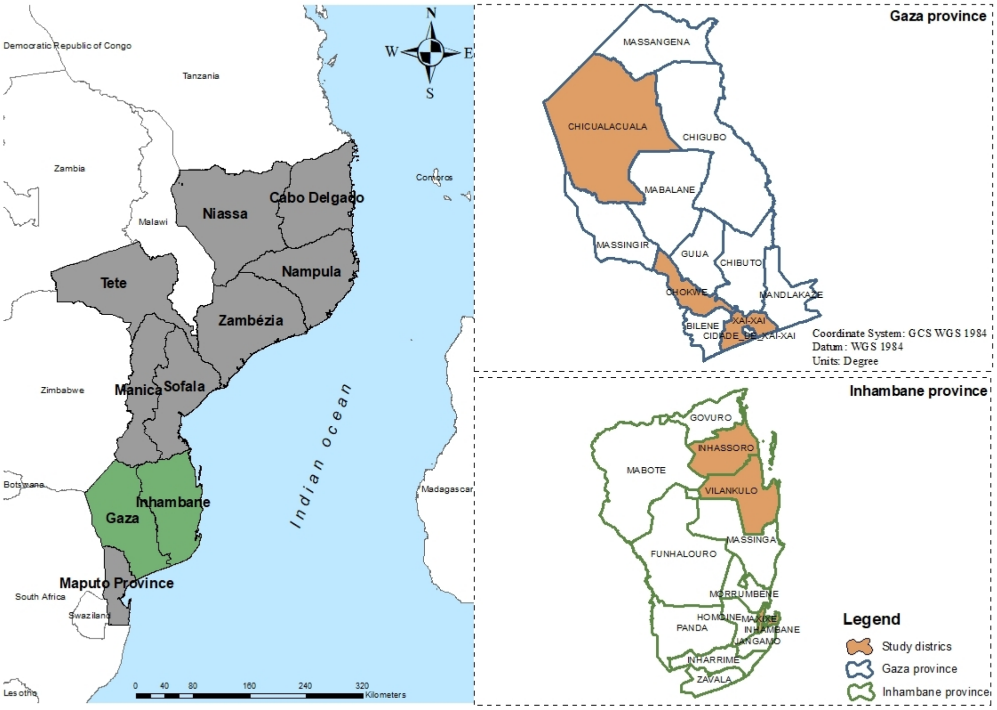

Figure 1: Map showing the study provinces and districts.

297x210mm (96 x 96 DPI)

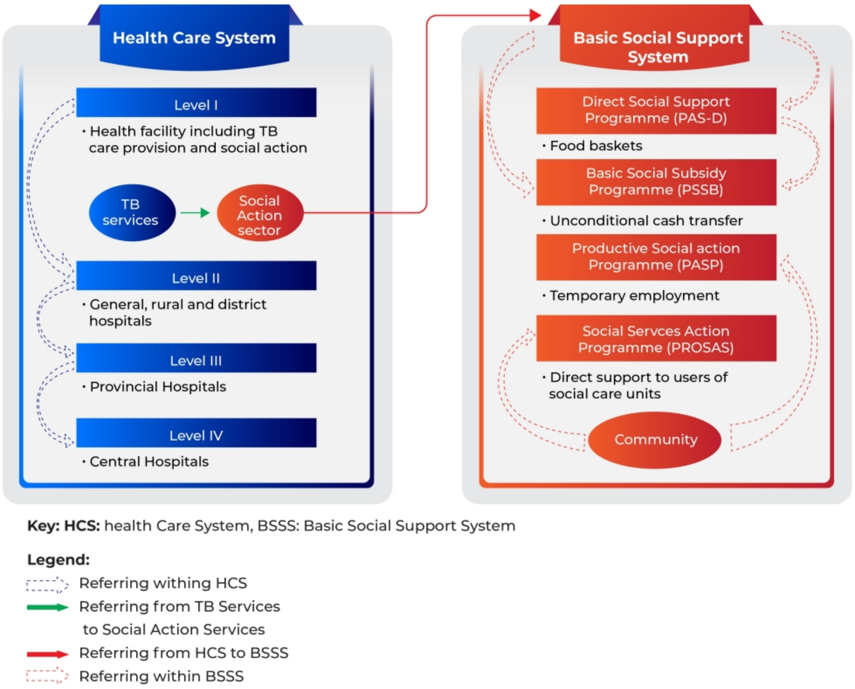

Figure 2: Current structure of Healthcare and Basic Social Support System. The black arrow represents the referral from the Social Action Sector, a representation of the National Institute of Social Action (INAS) within the health facility with the responsibility of preparing the documents for support requests, to the Basic Social Support System at INAS.

333x245mm (300 x 300 DPI)

Coordination of HEalth and Social care for TB patients in Mozambique: Policy dialogue and situation analysis - CHEST ENGLISH_**DECISION MAKERS**

Participant ID:______________

SCREENING BY RESEARCH TEAM:

Thank you for speaking with me_________________ regarding the "**Coordination of HEalth and Social care for TB patients in Mozambique**" study. This project is being led by researchers from [Karolinska Institute from Sweden and Instituto Nacional de Saúde from Mozambique]

I would like to ask you a few questions in order to determine whether you may be eligible for the research. Before I begin the screening I would like to tell you a little bit about the research. The purpose of this research is to create evidence of feasible and locally-appropriate social support scheme and strengthen understanding of the socioeconomic impact of TB in Mozambique.

Would you like to continue with the screening? The screening will take approximately one minute. You do not have to answer any questions you do not wish to answer or are uncomfortable answering, and you may stop at any time. Your participation in the screening is voluntary. If you do not qualify for the study, your answers to the screening will be destroyed. If you qualify for the research, decide to participate and agree to the informed consent, the answers to the screening will be only accessible to the research team.

Would you like to continue?

[*If no, thank the person and conclude the interview*]
[*If yes, continue with the IFC*]

For peer review only - http://bmjopen.bmj.com/site/about/guidelines.xhtml

Coordination of HEalth and Social care for TB patients in Mozambique: Policy dialogue and situation analysis - CHEST ENGLISH_**DECISION MAKERS**

Open- Ended Questions

Note: The in-depth interview will be open-ended and guided by the respondent's answers. This outline reflects a general guide for the in-depth interviews.

The interviews are meant to help us understand what could be the better socio economic support scheme for TB patient in Mozambique. We are particularly interested in understanding how you financial status changes after TB diagnose and what kind of social support scheme you value most. Actual questions asked during the interview will vary based on participant responses.

SCRIPT: Now I'd like to talk to you about your experience with TB social support services

BACKGROUND

1. Please tell me of your experience in this (health/social) service?
2. What are your main responsibilities?
3. What are the key decisions or activities that your service undertakes?

TUBERCULOSIS SUPPORT

4. Does your service deal with people who have tuberculosis? *If yes, how? If no, why not?*
5. Are you aware of support available to TB patients beyond medication? *Please describe. Does your organization do this? What other organizations are involved?*
6. What kind of support do you think TB patients need most?
7. What are the main challenges facing TB patients? What can/does your organization do about these?

INTERVENTIONS AND INTERSECTORAL WORK

1. To what extent does your organization work intersectorally (with another government department, health/social)?
2. Do they work together to support TB patients? If yes, how? If not, why not?
3. What would be a good way of collaborating with other departments for supporting TB patients? What could be done? Whose responsibility should it be to collaborate?
4. What kind of intervention in your opinion would be suitable to supporting TB patients economically and socially? What should they get, and whose responsibilities would this be?

For peer review only - http://bmjopen.bmj.com/site/about/guidelines.xhtml

Themes	Category	Sub-category	Codes
**(1) Policymakers suggested several solutions but thought that the implementation of many of them would be limited by available resources**	Benefits of supporting people with TB	–	–
	Incurred costs and mitigation	Cost mitigation	Coping mechanisms
			Current mitigation activities
			Provision entity
			Types of strategies for cost mitigation
		Financial impact	Impact on overall economy
			Ability to cope with the TB-related cost
			Impact on the course of disease
			Impact on families' lives
			Impact on patients' lives
		Type of incurred cost	During care seeking
			During treatment
			Factor influencing the occurrence of cost
	Potential solutions for addressing costs	Challenges faced by people with TB	Bottlenecks of TB treatments
			Problems faced by people with TB
			Problems faced by patients' families
		Source of support provision	Availability of support
			Current institutional actions
		Type of needed support	Better housing
			Challenges of receiving direct cash
			Children support / Education for children
			Community actors
			Easy access and expansion of good quality health service
			Home visits through deployment of health workers
			Psychological support
			Cash
			Food
			Transport
**(2) Lack of shared views or processes related to intersectoral collaboration between health and social protection sector hinders design and implementation of social support for people with TB**	Existing collaboration with other sectors	Challenges of collaborations	Barriers
			Facilitators
		Existing collaborations with other sectors	Key stakeholders currently left aside
			Involved stakeholders
			Mechanisms of collaborations and referrals of people with TB
			Type of collaborating institutions

Table 3: Categories and codes created during the coding process on Dedoose software, and the themes generated during subsequent analysis

COREQ (COnsolidated criteria for REporting Qualitative research) Checklist

A checklist of items that should be included in reports of qualitative research. You must report the page number in your manuscript where you consider each of the items listed in this checklist. If you have not included this information, either revise your manuscript accordingly before submitting or note N/A.

Topic	Item No.	Guide Questions/Description	Reported on Page No.
**Domain 1: Research team and reflexivity**			
Personal characteristics			
Interviewer/facilitator	1	Which author/s conducted the interview or focus group?	
Credentials	2	What were the researcher's credentials? E.g. PhD, MD	
Occupation	3	What was their occupation at the time of the study?	
Gender	4	Was the researcher male or female?	
Experience and training	5	What experience or training did the researcher have?	
Relationship with participants			
Relationship established	6	Was a relationship established prior to study commencement?	
Participant knowledge of the interviewer	7	What did the participants know about the researcher? e.g. personal goals, reasons for doing the research	
Interviewer characteristics	8	What characteristics were reported about the inter viewer/facilitator? e.g. Bias, assumptions, reasons and interests in the research topic	
**Domain 2: Study design**			
Theoretical framework			
Methodological orientation and Theory	9	What methodological orientation was stated to underpin the study? e.g. grounded theory, discourse analysis, ethnography, phenomenology, content analysis	
Participant selection			
Sampling	10	How were participants selected? e.g. purposive, convenience, consecutive, snowball	
Method of approach	11	How were participants approached? e.g. face-to-face, telephone, mail, email	
Sample size	12	How many participants were in the study?	
Non-participation	13	How many people refused to participate or dropped out? Reasons?	
Setting			
Setting of data collection	14	Where was the data collected? e.g. home, clinic, workplace	
Presence of non-participants	15	Was anyone else present besides the participants and researchers?	
Description of sample	16	What are the important characteristics of the sample? e.g. demographic data, date	
Data collection			
Interview guide	17	Were questions, prompts, guides provided by the authors? Was it pilot tested?	
Repeat interviews	18	Were repeat inter views carried out? If yes, how many?	
Audio/visual recording	19	Did the research use audio or visual recording to collect the data?	
Field notes	20	Were field notes made during and/or after the inter view or focus group?	
Duration	21	What was the duration of the inter views or focus group?	
Data saturation	22	Was data saturation discussed?	
Transcripts returned	23	Were transcripts returned to participants for comment and/or	

For peer review only

Topic	Item No.	Guide Questions/Description	Reported on Page No.
		correction?	
**Domain 3: analysis and findings**			
Data analysis			
Number of data coders	24	How many data coders coded the data?	
Description of the coding tree	25	Did authors provide a description of the coding tree?	
Derivation of themes	26	Were themes identified in advance or derived from the data?	
Software	27	What software, if applicable, was used to manage the data?	
Participant checking	28	Did participants provide feedback on the findings?	
Reporting			
Quotations presented	29	Were participant quotations presented to illustrate the themes/findings? Was each quotation identified? e.g. participant number	
Data and findings consistent	30	Was there consistency between the data presented and the findings?	
Clarity of major themes	31	Were major themes clearly presented in the findings?	
Clarity of minor themes	32	Is there a description of diverse cases or discussion of minor themes?	

Developed from: Tong A, Sainsbury P, Craig J. Consolidated criteria for reporting qualitative research (COREQ): a 32-item checklist for interviews and focus groups. *International Journal for Quality in Health Care*. 2007. Volume 19, Number 6: pp. 349 – 357

Once you have completed this checklist, please save a copy and upload it as part of your submission. DO NOT include this checklist as part of the main manuscript document. It must be uploaded as a separate file.

For peer review only
